# Polish is quantitatively different on quartzite flakes used on different worked materials

**DOI:** 10.1371/journal.pone.0243295

**Published:** 2020-12-03

**Authors:** Antonella Pedergnana, Ivan Calandra, Adrian A. Evans, Konstantin Bob, Andreas Hildebrandt, Andreu Ollé

**Affiliations:** 1 TraCEr, Laboratory for Traceology and Controlled Experiments at MONREPOS Archaeological Research Centre and Museum for Human Behavioural Evolution, RGZM, Neuwied, Germany; 2 School of Life Sciences, University of Bradford, Bradford, West Yorkshire, United Kingdom; 3 Scientifc Computing and Bioinformatics, Institute of Computer Science, Johannes Gutenberg University, Mainz, Germany; 4 IPHES, Institut Català de Paleoecologia Humana i Evolució Social, Tarragona, Spain; 5 Departament d’Història i Història de l’Art, Universitat Rovira i Virgili, Tarragona, Spain; Universita degli Studi di Ferrara, ITALY

## Abstract

Metrology has been successfully used in the last decade to quantify use-wear on stone tools. Such techniques have been mostly applied to fine-grained rocks (chert), while studies on coarse-grained raw materials have been relatively infrequent. In this study, confocal microscopy was employed to investigate polished surfaces on a coarse-grained lithology, quartzite. Wear originating from contact with five different worked materials were classified in a data-driven approach using machine learning. Two different classifiers, a decision tree and a support-vector machine, were used to assign the different textures to a worked material based on a selected number of parameters (*Mean density of furrows*, *Mean depth of furrows*, *Core material volume-Vmc*). The method proved successful, presenting high scores for bone and hide (100%). The obtained classification rates are satisfactory for the other worked materials, with the only exception of cane, which shows overlaps with other materials. Although the results presented here are preliminary, they can be used to develop future studies on quartzite including enlarged sample sizes.

## Introduction

Quantification of use-wear has recently seen an increasing interest among specialists [1, 2 and references therein]. Use-wear studies using metrology can provide a robust and quantitative approach to analysis, and they have the potential to improve and complement previous qualitative methodologies, which have performed poorly in blind-tests [3–6]. Several techniques have been used to acquire 3D data in order to quantify use-wear, such as focus variation microscopy, laser profilometry, white-light interferometry and laser scanning confocal microscopy (LSCM) [1, 7–15]. Chert i.e. fine-grained silica sedimentary rocks, *sensu* [16], has been the most studied raw material in conventional use-wear studies which included large experimental datasets [e.g. 17–19]. Similarly, quantitative methods have mainly been applied on chert surfaces [7, 10, 12, 20, 21], with few attempts done to assess their potential for the analysis of coarse-grained rocks [22–24]. Trials on other raw materials, such as obsidian or basalt, have also been performed in the past [14, 25–27]. Quantitative surface analysis can be applied to materials other than rocks [2]. In fact, surfaces of ochre, bone and shells have also been analyzed mainly using confocal microscopy [28–31].

Nevertheless, quantification studies are still in their infancy and none of the tested techniques have systematically been incorporated into the domain of traceology [6]. Among the various techniques used to acquire 3D surface topographical data, data acquired with confocal microscopy proved to be able to discern contact materials obtained from experimentally produced polished surfaces on chert specimens [10, 12, 32]. LSCM was preferred over the other available techniques due to its ease of use, relatively quick acquisition time and inherent potential demonstrated by the initial studies that incorporated relatively small datasets [20, 22, 33, but see 34]. Confocal microscopes are generally coupled with optical microscopes, which are useful for observing areas to be measured [12, 35, 36]. 3D topographies are generally acquired to provide quantitative data of the worn areas resulting from contact with different materials. The main underlying goal of doing this is to limit the analysts’ subjectivity and to increase the general accuracy of the method [6, 37]. Moreover, it improves repeatability and reproducibility of the analyses [38]. However, it has been shown that it is not yet possible to automatically locate and isolate the worn areas (i.e. areas of interest) for analysis [20], implying that the choice of the area to be analyzed is still subject to the analyst’s discretion. In this regard it complements the ‘traditional’ microwear method in that the one aspect that has performed well in prior blind testing is the expert analyst’s ability to identify the location of wear [6].

A further reason that explains the high investment of energy and time into developing and refining quantitative methods in use-wear analysis is the possibility of producing probability statements based on surface parameters and the use of a variety of statistical methods [12, 32]. Researchers were able to distinguish polished areas formed after contact with a variable number (two to six) of worked materials [10, 21, 32]. Moreover, different humidity content of the contact materials has proved to produce different polishes that can be differentiated based on confocal measurements (e.g. wild vs. domestic cereals) [39, 40]. All this contributed to give confocal microscopy its prominent role in use-wear studies involving metrological applications.

Coarse-grained rocks such as quartzite have been less frequently investigated in conventional use-wear studies [41–43 and references therein], as well as in quantitative ones [22, 23]. As a consequence, comparable quantitative datasets of these rocks are quite limited. Yet, quartzite is one of the most frequently employed materials for producing stone tools throughout the Plio-Pleistocene worldwide [e.g. 44–47]. For instance, it is very abundant in lithic assemblages of key-sites for the understanding of human evolution, such as the Atapuerca [48, 49] and Olduvai [44, 50, 51] sites. Hence, it is crucial to apply quantitative tests on this type of rock to be able to better characterize, and therefore recognize, different use-wear traits on such surfaces. It is very likely that, in the same way that some qualitative features are more useful than others to describe use-wear on different raw materials, different sets of surface parameters might prove more powerful than others for quantitatively discriminating worn surfaces on different rocks. In the attempt to find out more about the most suitable parameters for each raw material, trial and error experiments are necessary. Moreover, it is important to consider a number of varieties of the same rock when setting up future experiments to test quantitative methods, knowing that they can wear down differently.

This paper presents the first metrological study of quartzite flakes used on five contact materials documented with LSCM. Two different varieties of quartzite were included in the experiments. One of the main aims of this study was to add quantitative 3D data to the visual descriptions of polished areas on quartzite tools provided in previous contributions [36, 41–43]. Moreover, the quantification of worn areas produced after contact with a number of worked materials commonly included in reference collections will contribute to our general understanding of the polishing process on quartzite.

## Materials and methods

### Experimental design

**Fig 1:**
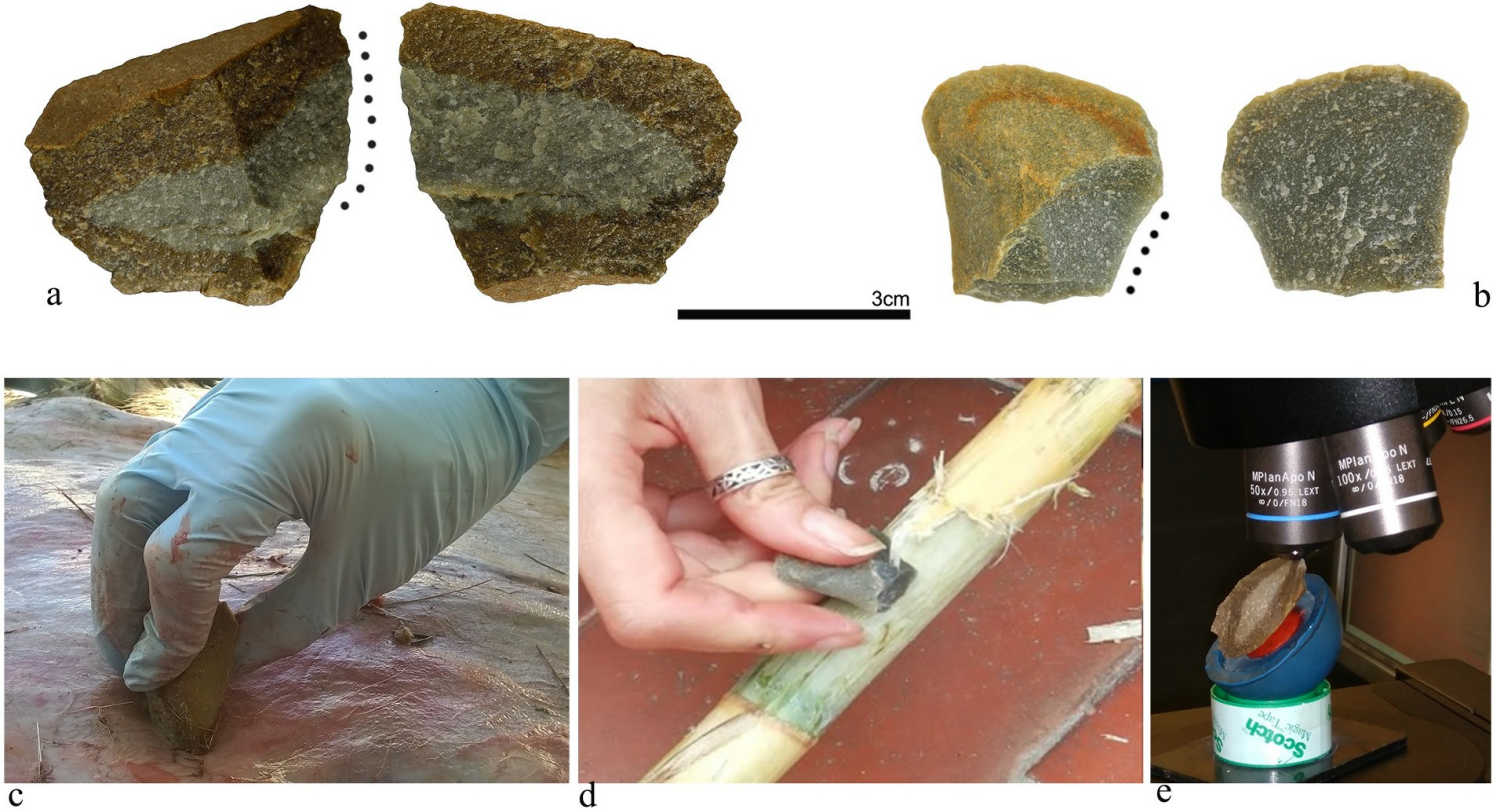
The experimental design. a) VSH4-6; b) A35-2; c) VSH4-6 used to scrape fresh deer skin; d) A35-2 used to whittle a fresh cane stem; e) One of the VSH variety samples under the confocal microscope during data acquisition.

In order to limit raw-material intra-variability, we only included one cobble for each of the two distinct quartzite varieties in our experimental program. The cobbles were collected in Northern Spain, in the vicinity of the Sierra de Atapuerca archaeological complex. The two varieties are named for their provenance: VHS—Villasur de Herreros and A3 –at the 3rd kilometer of the BU-820 road (Fig 1a and 1b). The added number following the general denomination of the variety indicates the number of the selected cobble (VSH4 and A35). These varieties were selected in an attempt of constructing a large reference collection of use-wear produced on numerous varieties of quartzite coming from Northern Spain and Southern France [41, 43, 52, 53]. This reference collection was aimed at interpreting the archaeological assemblages of Gran Dolina-TD10 [48, 54, 55] and Payre [56]. Fourteen experimental unretouched flakes were knapped and each one was used on a single worked material (Table 1). Two experiments were carried out in a second step at the IPHES’s lab (samples A35-3 and 7) and subsequently sent to Bradford for data acquisition. Unfortunately, these two samples were damaged after mailing them and therefore they could not be scanned.

**Table 1:** The fourteen experimental samples presented in this study, sorted into the two different varieties of quartzite: VSH and A3. The worked materials included in the experiments are listed as well as the type of action (all unidirectional movements).

N.	Reference	Worked material type and state	Action	Analyzed herein
1	VSH4-1	Soaked antler	Whittling	Y
2	VSH4-2	Fresh cane	Whittling	Y
3	VSH4-3	Dry skin	Scraping	Y
4	VSH4-4	Fresh wood	Whittling	Y
5	VSH4-5	Fresh bone	Whittling	Y
6	VSH4-6	Fresh skin	Scraping	Y
7	VSH4-7	Unused	-	Y
8	A35-1	Soaked antler	Whittling	Y
9	A35-2	Fresh cane	Whittling	Y
10	A35-3	Dry skin	Scraping	Y
11	A35-4	Fresh wood	Whittling	Y
12	A35-5	Unused	-	Y
13	A35-6	Fresh skin	Scraping	N
14	A35-7	Fresh bone	Whittling	N

Five different worked materials commonly associated with early prehistoric tasks—wood, bone, antler, fresh and dry skin, and cane were worked for an hour. The worked materials comprised a type of softwood, Aleppo pine (*Pinus halepensis*), a red deer antler (*Cervus elaphus*), long bovid bones (*Bos taurus*) and stems of giant cane (*Arundo donax*). All materials were worked in a fresh state, except for the dry skin (*Cervus elaphus*). The antler was soaked for 48 hours in water before the experiment.

The movement was limited to whittling/scraping in order to control variables that may impact polish development (Fig 1c and 1d). Both are transversal movements where the used edge is held at an approximately right angle to the direction of use [17]. In whittling actions, the working angle is very low (in our cases 40° < α < 20°) and the movement is a pushing away motion whereas for scraping ones, the working angle is always higher (in our cases 40° > α >70°) and the direction of the movement is a- pulling toward motion All the experiments were performed by one of the authors (A.P.), in order to maintain all the variables involved (e.g. the amount of exerted pressure, relative velocity, approximate number of strokes per min) as constant as possible. Single strokes were not counted, since the aim of the experiments was not to control polish development on different materials produced by the same number of strokes.

Knowing that polish formation generally takes longer to form on coarse-grained rocks than on fine-grained ones [22, 41, 57, 58], the length of the experiments was prolonged (60 min) to assure the formation of relatively large and well-developed polished areas. Two control samples (VSH4-7 and A35-5), one per variety, were left unused.

### Cleaning protocol

Soon after the conclusion of the experiments, all the used flakes were cleaned in order to remove residues of the contact materials. They were initially soaked in water and then subjected to several ultrasonic baths: 1. Bath in hydrogen peroxide (H_2_O_2_, 10 Vol) for 15min. This was done to remove organic matter; 2. Bath in a neutral detergent solution (^®^Derquim- LM02) for 15min to remove remnants of organic matter; 3. Bath in acetone (technical grade) for 5 min to remove handling residues. At this point, worn surfaces were documented with scanning electron microscopy at the Rovira i Virgili University (Tarragona) (JEOL JSM-6400; FEI quanta 600 SEM) before acquiring 3D data [36].

Because some months passed between the two analyses and all lithics were either manually transported or mailed to the laboratory at Bradford, a second cleaning procedure was applied.

Immediately before analysis with LSCM at the University of Bradford, the flakes were soaked in a 10% NaOH (sodium hydroxide) solution for 10 min to eliminate all possible residues deriving either from accidental handling since the SEM observations or from the plastic bags where they were stored until analysis. A last bath in water for 10 min was necessary to eliminate all residues of the sodium hydroxide solution. Surfaces of interest were additionally rinsed with chromatography grade ethanol to remove dust particles and dried immediately before placing them under the confocal microscope.

### 3D data acquisition

Scans were acquired on well-developed polished areas. Randomly selected areas were measured on the unused samples using the same settings.

The microscope used was an Olympus LEXT OLS4000 laser scanning confocal microscope (LSCM), located at the School of Life Sciences, at the University of Bradford (Fig 1e). Scans were acquired using the 50x objective at 1x zoom (MPLAPONLEXT50-1x; NA = 0.95) with a field of view of 256 x 256 μm and a frame size of 1024 x 1024 pixels. The laser source has a wavelength of 405 nm. The step size was set to 10 nm.

The resulting outputs were *.*lext* files including height map (topography), maximum intensity map and brightfield images as layers (S1a–S1c Fig).

### 3D surface texture analysis

#### Overview

Each acquired height map (topography), commonly called scan, was processed in batch using templates. These templates applied different operations and filters in order to make the calculation of 3D surface texture parameters possible.

Because the polished areas were smaller than the field of view, we extracted two 50 x 50 μm sub-areas from each scan; this step was the only manual step in the whole workflow. We then compensated for (local) tilt of the sub-areas by leveling. Because we focus on the texture, we removed the noise and the form (see section “Workflow and terminology” below for definitions). We then cleaned the sub-areas from defects by removing outliers and thresholding. Removing these defects results in missing data (non-measured points), which prevent the calculation of some parameters. These non-measured points were therefore filled before the calculation of the 3D surface texture parameters.

Parameters from five types of analysis were calculated: ISO 25178–2 [59], scale-sensitive fractal analysis [60, 61], furrow analysis, texture isotropy and texture direction.

#### Technical details

Data analyses were conducted using ConfoMap (version 7.4.8633) (a derivative of MountainsMap Imaging Topography developed by Digital Surf, Besançon, France). Two templates were created, following previous publications [10, 62].

A first template was created:

1. For each scan (n = 43), two 50 x 50 μm sub-areas were extracted (Table 2);
2. For each sub-area (n = 86) the topography layer was extracted and saved as SUR files;

**Table 2:** Number of scans per each sample and of the related extracted sub-areas.

Sample reference	Number of scans	Extracted sub-areas– 50x50 μm
VSH4-1	4	8
VSH4-2	5	10
VSH4-3	3	6
VSH4-4	2	4
VSH4-5	5	10
VSH4-6	5	10
VSH4-7	1	2
A3-1	2	4
A3-2	3	6
A3-4	3	6
A3-5	3	6
A3-6	3	6
	**TOT = 39**	**TOT = 78**

The areas of interest were manually extracted from well-polished surfaces following past published works [10, 32] (S1d–S1g and S2 Figs);

A second template was created to process all extracted 3D sub-areas (Table 2). The analysis workflow was as follows (S1h Fig):

1. *Level*: LS plane by subtraction;
2. *Remove form*: polynomial degree 3;
3. *Remove outliers*: maximum slope allowed 80°, soft method, remove measurement noise, and non-measured points not filled-in;
4. *Threshold*: *0*.*1% to 99*.*9%*, reference = height from mean plane, set as non-measured points
5. *Robust Gaussian filter* to remove the noise: 2.5 μm, manage end effects, keep “waviness”;
6. *Fill in non-measured points*: replace by a smooth shape calculated from the neighbours (S1i and S1j Fig);
7. Calculate ISO 25178–2 [59] + SSFA [60, 61] + furrow analysis + texture isotropy + texture direction parameters.

All ConfoMap templates for each surface (sub-area) in MNT and PDF formats (including all original and processed surfaces, as well as all results) are freely available on Zenodo (https://doi.org/10.5281/zenodo.3979116).

We identified issues with some sub-areas (Table 3), which are ultimately due to the properties of quartzite. During scanning, care was taken so that the whole scanned surface is as horizontal as possible. However, the sub-areas represent only a small portion of these surfaces, and some sub-areas were therefore acquired with a strong local tilt. Together with the large topographic variations typical for quartzite, this large tilt can lead to the erroneous measurement of some points, usually in the deepest areas. These points were later removed by the *Remove outliers* operator, leaving substantial areas of non-measured points to be later filled in by the *Fill in non-measured points* operator. The result is that these usually deep parts of the problematic sub-areas were completely filled in, artificially increasing the values of the height and volume parameters.

**Table 3:** Potientially problematic sub-areas.

Sample reference	Point number	Area number
VSH4-1	2	2
VSH4-1	3b	1
VSH4-2	3a	1
VSH4-2	3b	2
VSH4-4	1b	2
VSH4-5	1b	1
VSH4-5	1c	1
VSH4-5	1c	2
		**TOT = 8**

Additionally, some sub-areas were identified as potentially problematic because they go over the edges of quartz grains. At the edges, the topography can be very steep and this too can result in the erroneous measurement of some points, ultimately artificially increasing the values of the height and volume parameters.

Because these issues cannot be completely avoided on quartzite, we decided to run the analyses described below with and without these potentially problematic sub-areas.

#### Workflow and terminology

In the present study, we developed an analysis workflow by combining and adapting approaches of previous studies. We tried to follow Evans et al. [20] because the data were acquired on the same LSCM, but we argue that extra processing steps were necessary. In particular, we incorporated some of the steps of Arman et al. [62], who developed a template to reduce inter-microscope variability in dental microwear texture analysis. Our analyzed surfaces (sub-areas) are 50 x 50 μm, as in Evans et al. [20]. The optical lateral resolution of the 50x/0.95 objective is high (see Supplementary Material 1 of [38] but the digital resolution (= pixel size) is too low (0.25 μm) to allow smaller areas to be analyzed. On the other hand, larger areas do not fit on the quartz grains of these quartzite varieties. We, therefore, extracted two sub-areas per scan. Note, however, that Ramadarshan et al. [63] showed that, for dental microwear texture analysis, one large area is better for discrimination than several smaller areas covering the same total area.

Our template first leveled and removed the form following Arman et al. [62], although we used a polynomial of degree 3, which seemed more appropriate for our data. Then, still following Arman et al. [62], we removed outliers and thresholded the surfaces to remove aberrant points (spikes due to measurement errors). Since our digital resolution is already low (pixel size = 0.25 μm), there was no need to resample as recommended by Arman et al. [62], who resampled to 0.2 μm. This might change when acquiring with other confocal microscopes featuring higher digital resolutions, if one wants to compare data. We followed Evans et al. [20] for the application of the robust Gaussian filter. This filter, with a cut-off at 2.5 μm, is meant to remove the measurement noise from the surface [following 20, 62] and ISO 4287/4288 [64, 65].

However, we kept both the roughness and the waviness since it is not clear yet which scale is the most relevant and since the SSFA is applied across the scales. The final step of our workflow was to fill-in non-measured points following Arman et al. [62]. Indeed, this is necessary to calculate the SSFA and some ISO parameters.

A note on terminology is warranted here to avoid confusion. Following Leach [66], the term surface *topography* describes the overall surface structure, while surface *form* is defined as the shape of the sampled area, and surface *texture* is what remains when the form is removed from the topography during analysis. In this sense, texture includes both *roughness* and *waviness*. The limits between these terms are based on pre-defined wavelength cut-offs. These definitions differ from those of Evans et al. [20], where texture includes only the roughness component and where topography and waviness are synonyms. To add to the confusion, the terms roughness and waviness can also be independently related to the application of a filter. For the sake of the argument, let us first call the roughness and waviness mentioned above “absolute roughness/waviness”.

When applying a filter in MountainsMap 7 using the "standard filter" operator, the surface containing the wavelengths smaller than the cut-off is called “roughness”, while the surface containing the wavelengths larger than the cut-off is called “waviness”, irrespective of the absolute wavelength. Let us refer to these as “relative roughness/waviness”. To illustrate this, the cut-off at 2.5 μm for our robust Gaussian filter is situated at the limit between (absolute) instrument noise and texture (also called micro- or nano-roughness) (i.e. absolute roughness + waviness, the form having been removed before). Because we wanted to remove the noise, we excluded wavelengths smaller than the cut-off and kept the wavelengths larger than the cut-off. These larger wavelengths are referred to as “waviness” in the software (i.e. relative waviness), which is why the step #6 of the history of operators in our templates (see S1h Fig and "processing-quartzite-final" templates at https://doi.org/10.5281/zenodo.3979116) shows “waviness”, even though it actually includes both absolute roughness and waviness.

MountainsMap 8 changed its terminology to avoid this confusion. There is now a special operator called “S-filter (λs)” that “remove[s] the micro-roughness”, while the “metrological filter” is used to “separate roughness […] and waviness […] components” (tooltips for these operators in MountainsMap 8).

Clearly, a lot remains to be done on defining an appropriate analysis workflow for lithics in general.

### Statistical analysis

Preparation of the data and all descriptive analyses (summary statistics and plots) were performed in the open-source software R (v. 3.6.3; [67]) through RStudio (v. 1.2.5042; [68]) for Microsoft Windows 10. The following packages were used: chron (v. 2.3–55; [69]), doBy (v. 4.6.5; [70]), ggConvexHull (v. 0.1.0; [71]), ggplot2 (v. 3.3.0; [72]), openxlsx (v. 4.1.4; [73]), and R.utils (v. 2.9.2; [74]). Scripts, results and reports of the analyses in HTML format, created with knitr (v. 1.28; [75–77]) and rmarkdown (v. 2.1; [78]; v. 2.0; [79]), are freely available on Zenodo (https://doi.org/10.5281/zenodo.3979139).

#### Datasets

As explained above, the same analysis was run on two datasets: one containing the data from all processed sub-areas (“full dataset” hereafter, n = 78), and the second excluding the data from the potentially problematic sub-areas (“restricted dataset” hereafter, n = 70; Table 3). The parameters of the analyses were adjusted as required by the data (see below and supplementary data on Zenodo; https://doi.org/10.5281/zenodo.4249219).

#### Handling missing data

Some parameters could not be calculated on all surfaces leading to missing values (see table "processing-quartzite-final.xlsx" on Zenodo (https://doi.org/10.5281/zenodo.3979139). The machine learning models we applied cannot deal with missing input, so cases where parameters values could not be calculated (missing data) were resolved as follows.

In the case that there was only one value missing for a given group, i.e. combination of quartzite type and worked material, the missing value was filled by the median of the other values in the group; otherwise the parameter was discarded for further analysis. This way, the parameters *Asfc* and *Smfc* (full dataset) and *HAsfc9* (both datasets) were made useable for further analysis, while the parameters *HAsfc81*, *Periodicity*, *Period* and *Direction*.*of*.*period* had to be discarded for both datasets. After the data preprocessing, 33 parameters and the quartzite type were available for every scan on both datasets.

#### Machine learning: Data split, feature selection and classification

*Overview*. As discussed above, some parameters could not be calculated on some surfaces, leading to missing data. The machine learning techniques applied cannot deal with missing data, so we excluded these parameters from subsequent analyses. Among all possible acquired data that one could feed to the machine learning models, we selected only those with the highest predictive power based on the amount of information they provide on the worked materials. Even though all data could potentially provide relevant information, including information with low predictive power typically lowers the performance of a machine learning model. Using these meaningful independent, input data (i.e. certain parameters and possibly the quartzite type) and their corresponding dependent, output data (i.e. type of worked material), we now try to find a generalizable mapping between those input and output data. This is achieved by repeatedly adjusting numerical coefficients ("learning") of two types of certain mathematical formulas ("machines") that have been proven suitable in similar tasks: decision-tree classifiers and support-vector machines. The dataset is split into two: a training set used to define the mapping between input and output data, and a test set used to evaluate the accuracy and ability to perform on unseen input data. The results allow us to calculate the rate of correct classification for each worked material and for each method, given the chosen parameters and possibly the quartzite type.

*Technical details*. In the following, variables used to predict the worked material for each scan will be referred to as features and the type of worked material as the corresponding label. All subsequent analytical steps were conducted once using the 33 parameters and the quartzite type as features, and once using the 33 parameters without the quartzite type as a feature. The data was split into a test (33% of data, n = 26 for the full dataset and n = 24 for the restricted dataset) and a training set (remaining data, n = 52 for the full dataset and n = 46 for the restricted dataset) in a stratified fashion, to ensure roughly equal presence of quartzite types and labels in both test and training data.

**Fig 2:**
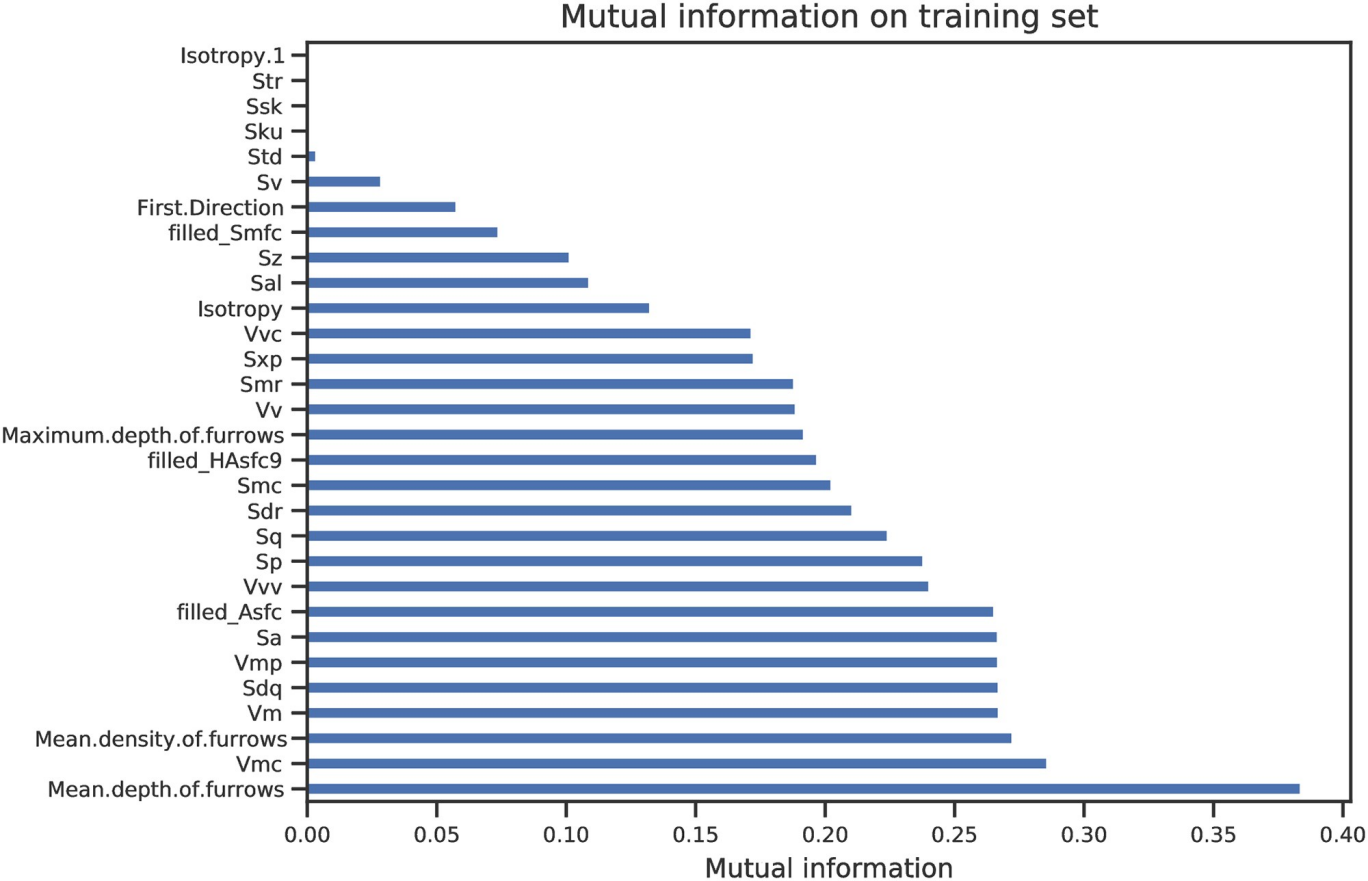
Mutual information on training set without type.

First, the predictive power of each feature is measured by the mutual information between the features and the labels. Mutual information [80] is a measure from the field of information theory and thus does not rely on the specific models used afterwards. Mutual information is a measure similar to correlation between two variables. A high value for one variable indicates that it explains well the second variable. In our case, the second variable is the worked material. So we chose variables with high mutual information because they tell us a lot about the worked materials in the dataset. In other words, the selected variables are expected to have a high predictive power. The subset of features that distinctly show higher mutual information than the rest of the features will be referred to as the *selected features* in the following text. As a test on the predictive power of the selected features and due to the general expectation that irrelevant features usually degrade the performance of a machine learning algorithm, the subsequent analyses were performed once with the full features set and once on the selected features. Thus, there were in total four test and training sets for each of the two datasets (full and restricted): using all 33 features or selected features together with quartzite type, and using all 33 features or selected features without considering quartzite type. When the quartzite type was not considered, a set of three features were selected: *Mean depth of furrows*, *Vmc (core material volume)*, *Mean density of furrows* (Fig 2). The same three features were selected when quartzite type was additionally considered a feature (S3 Fig).

Two common classification models were used: decision-tree classifiers with entropy-based splitting and support-vector machines (hereafter SVM) with different kernel functions. Both algorithms are based on the same principle. Each sample is described as a point in a (possibly) high-dimensional space with the values of each feature as its coordinates (S4 Fig). Separation of classes is achieved by drawing boundaries around regions of points with the same label in that so-called feature space. Decision-tree classifiers are restricted to boundaries orthogonal to a feature’s axis, i.e. setting a threshold on a single feature at a time. Support-vector machines, however, are allowed to use more flexible, i.e. non-orthogonal and curved, boundary shapes.

The respective hyperparameters, i.e. the maximum depth for the decision-trees and the kernel functions, the kernel coefficients, and the regularization parameters for the SVM, were optimized by 3-fold cross-validation on the training data using balanced accuracy as a performance measure. The balanced accuracy measures the fraction of correctly predicted instances, but accounts for imbalanced frequencies of classes. Thus, the classification is forced to work well on all classes instead of neglecting less frequent classes and concentrating on the most frequent ones [81].

The final models were trained on the full respective training data using the most promising hyperparameters from the above optimization procedure. Final performance evaluation was done on the test sets by measuring the balanced accuracy and computing normalized confusion matrices, i.e. the fraction of cases in which every pair of true label (i.e. actual worked material) and corresponding predicted label (i.e. worked material identified by the model) occurred.

The whole handling of missing data and the machine learning part were conducted using the following free and open-source software packages: pandas in version 0.25.0 [82], matplotlib in version 3.1.0 [83], numpy in version 1.17.0 [84] scikit-learn in version 0.22.2.post1 [85] and seaborn in version 0.9.0 [86] under the python programming language in version 3.7.3.

The full code and output are available on Zenodo (https://doi.org/10.5281/zenodo.4249219).

## Results

Two types of models were tested to process the raw data obtained with the confocal scans on the experimental sample: a decision tree and a support-vector machine (SVM).

### Dataset

The analyses on the two datasets (full and restricted) produce the same kind of results, but are very different in details, i.e. which parameters are selected by the mutual information criterion, the rates of classification for each worked material, etc. All results are available on Zenodo (https://doi.org/10.5281/zenodo.4249219) but only the results on the full dataset are presented here for two reasons. (1) The general classification rates are higher on the full dataset. This implies that the full dataset holds more potential for future studies. (2) Quartzite is a difficult material to scan with confocal microscopes and process for 3D surface texture analysis. It is therefore likely that other studies will stumble on the same difficulties. In other words, we argue that the potentially problematic sub-areas we identified do represent quartzite surfaces, with all their issues. Addressing these issues seems to be a fruitful avenue for future developments of the quantitative approach in microwear analysis.

### Selected features

All parameters (i.e. features) were tested both when the quartzite “type” (i.e., the rock variety, either VHS or A3) was considered as a feature and when it was excluded. The performance in classification rate is comparable when using either all features or the three selected features, whether quartzite type was considered as a feature or not (S5 Fig). Therefore, the selected features can be used without losing performance compared to using the full feature set, with the added benefit of a higher chance to work well on unseen data.

The three selected features, beside "type", are the ISO 25178–2 parameter core material volume (*Vmc*) and the parameters mean depth (*Mean depth of furrows*) and density (*Mean density of furrows*) of furrows. *Vmc* is the volume below the surface (i.e. *material volume*) when the highest 10% and lowest 20% of the points (i.e. *core*) are excluded [59, 87]. Furrows are micro-valleys. The furrow analysis identifies the areas where points are lower than the neighboring points on a given surface (Digital Surf, pers. comm. 2020). The mean density and depth of the identified furrows is then calculated.

### Quartzite type

**Fig 3:**
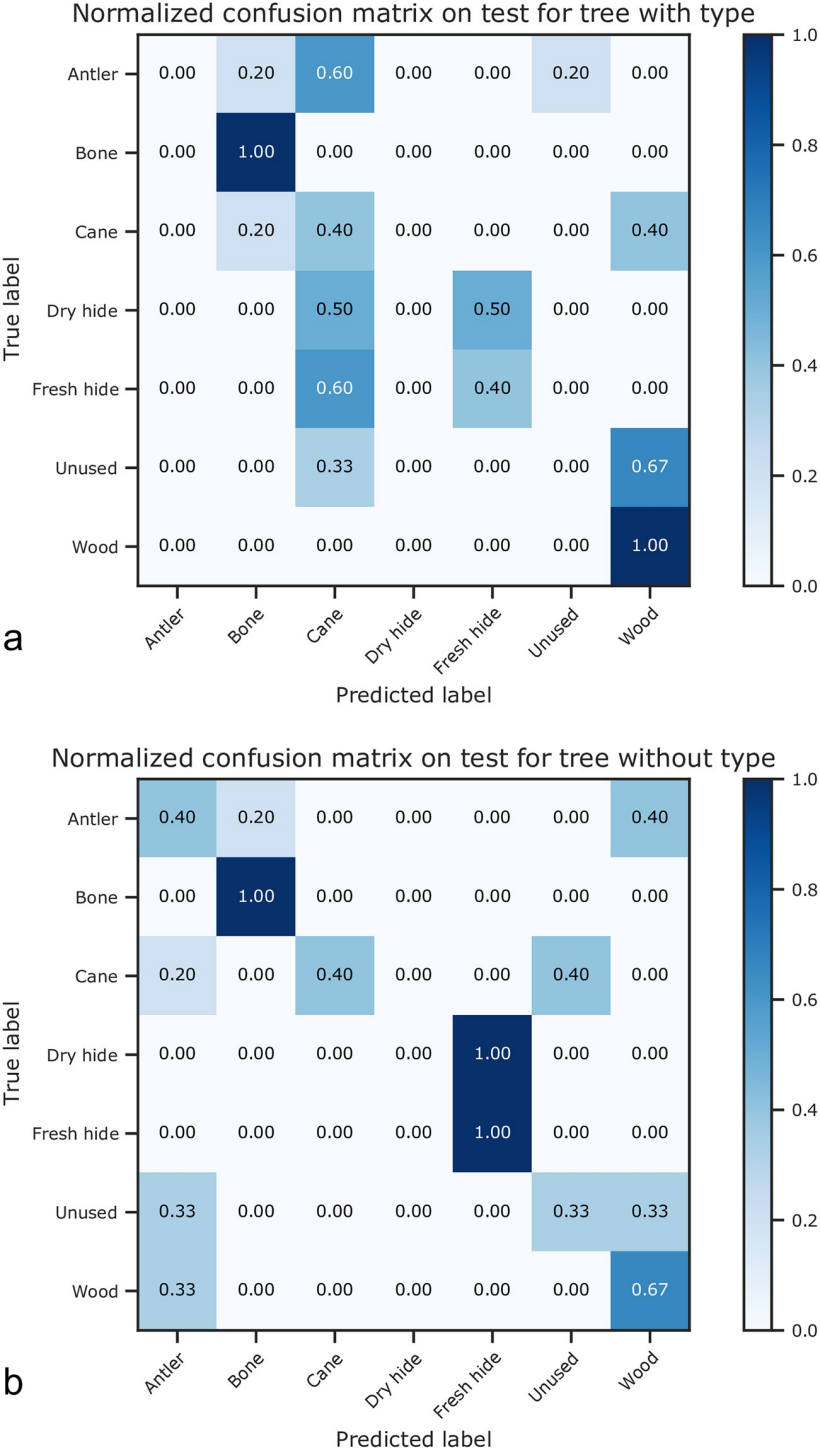
Normalized confusion matrices on the test set, both including (a) and discarding (b) the feature “type”. Non-normalized confusion matrices are freely available on Zenodo.

The overall classification rates are moderate for the selected features (the highest balanced accuracy score on the test set is found for the decision tree with type = 0.47), which is promising considering the small dataset and the large variation in classification rates between the different materials. For the decision tree, the classification rate is higher when the “type” is not taken into account in the analysis (balanced accuracy scores on the test set = 0.47 vs. 0.30). The normalized confusion matrices (with vs. without type) on the test set show the same trends (Fig 3). The only worked material that is better recognized when the “type” is considered is wood, while all other worked materials are equally or less correctly recognized (Fig 3).

This means that for the decision tree, the most general model has a higher success, being promising for future studies including larger datasets. This is not true for the SVM models. SVM performs much better when “type” is considered (balanced accuracy scores on the test set = 0.44 vs. 0.06), allowing, for example, discerning perfectly between fresh and dry hide (S6 Fig). When “type” is not included in this model, results are quite dispersive and most of the materials are not correctly discriminated (S7–S9 Figs).

**Fig 4:**
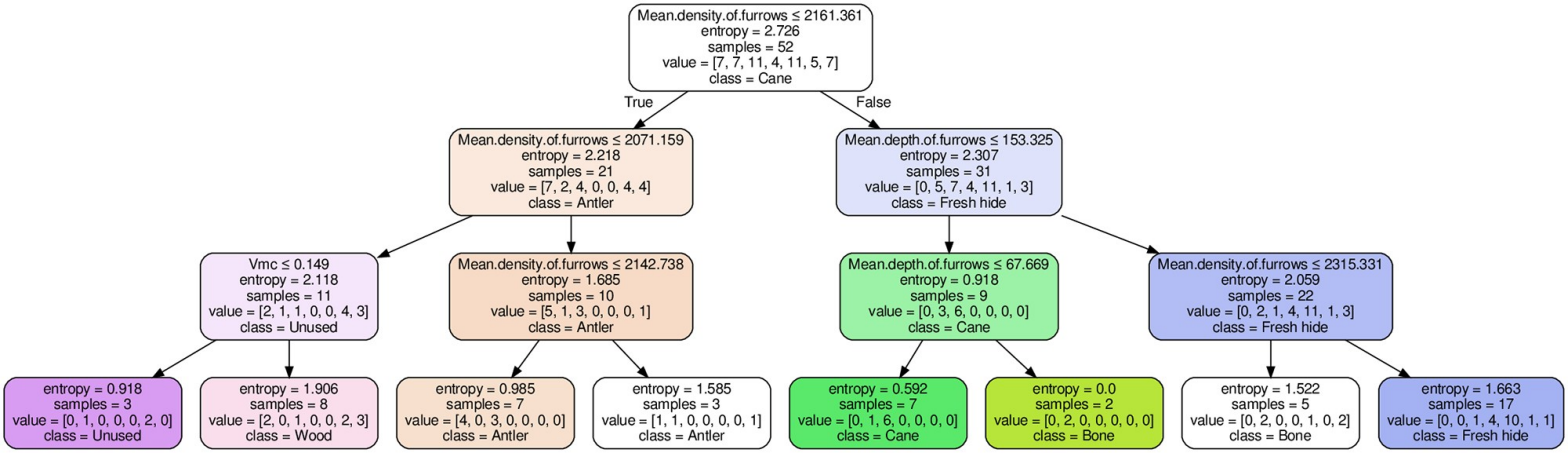
Decision-tree model without considering the quartzite variety (type).

Thus, it is reasonable to use the tree approach because of its better interpretability. Results for the decision tree on the test set with selected features not including type are described below. The tree itself is shown in Fig 4. The results for the decision tree including the type are found on Zenodo (https://doi.org/10.5281/zenodo.4249219), with the corresponding tree given in S10 Fig.

### Decision tree on test set, selected features, excluding type

Looking at the decision tree itself, each box has five attributes, one per line: (1) the parameter/feature, i.e. the quantity in question and the corresponding inequality; (2) the entropy of the splitting, 0 indicating pure groups; (3) the number of samples in the group, defined as the number of extracted sub-areas from the acquired 3D scans; (4) “value” is the list of samples per class (material category) number; and (5) “class” refers to the material category with most members in the sample. The class numbers correspond to the material categories in this order: 0 = Antler, 1 = Bone, 2 = Cane, 3 = Dry hide, 4 = Fresh hide, 5 = Unused, 6 = Wood.

On the test set, we see that bone and hide are perfectly classified (100%), although fresh and dry hide cannot be discriminated from one another (all are classified as fresh hide). Wood and antler, taken together, can be reliably discriminated from other materials but are not well discriminated from each other. Wood is correctly identified in the 67% of the cases, while it is misidentified as antler in the other cases. Antler is classified as either bone (20%) or wood (40%), and only in the 40% of the cases it is correctly identified. The unused samples cannot be discriminated from antler or wood (33% classification in each category). Cane is as likely to be classified as both unused and cane (40% each). It can also be often misclassified as antler (20%).

## Discussion

### Quantifying polish on quartzite

The use of confocal microscopy in use-wear analysis is relatively new and therefore, largerly unexplored [6]. For example, it has not been systematically employed in the study of coarse-grained rocks, such as quartzite [22, 23]. The potential to image use-wear on this type of rock has recently been shown [35, 36, 41]. The systematic documentation of the areas to be measured with the optical microscope mounted on the same confocal microscope used to acquire 3D surfaces allows analysts to carefully select the areas of interest. Moreover, this allows publishing the visual appearance of the measured surfaces, therefore contributing to transparency and data comparison (S2 Fig).

**Fig 5:**
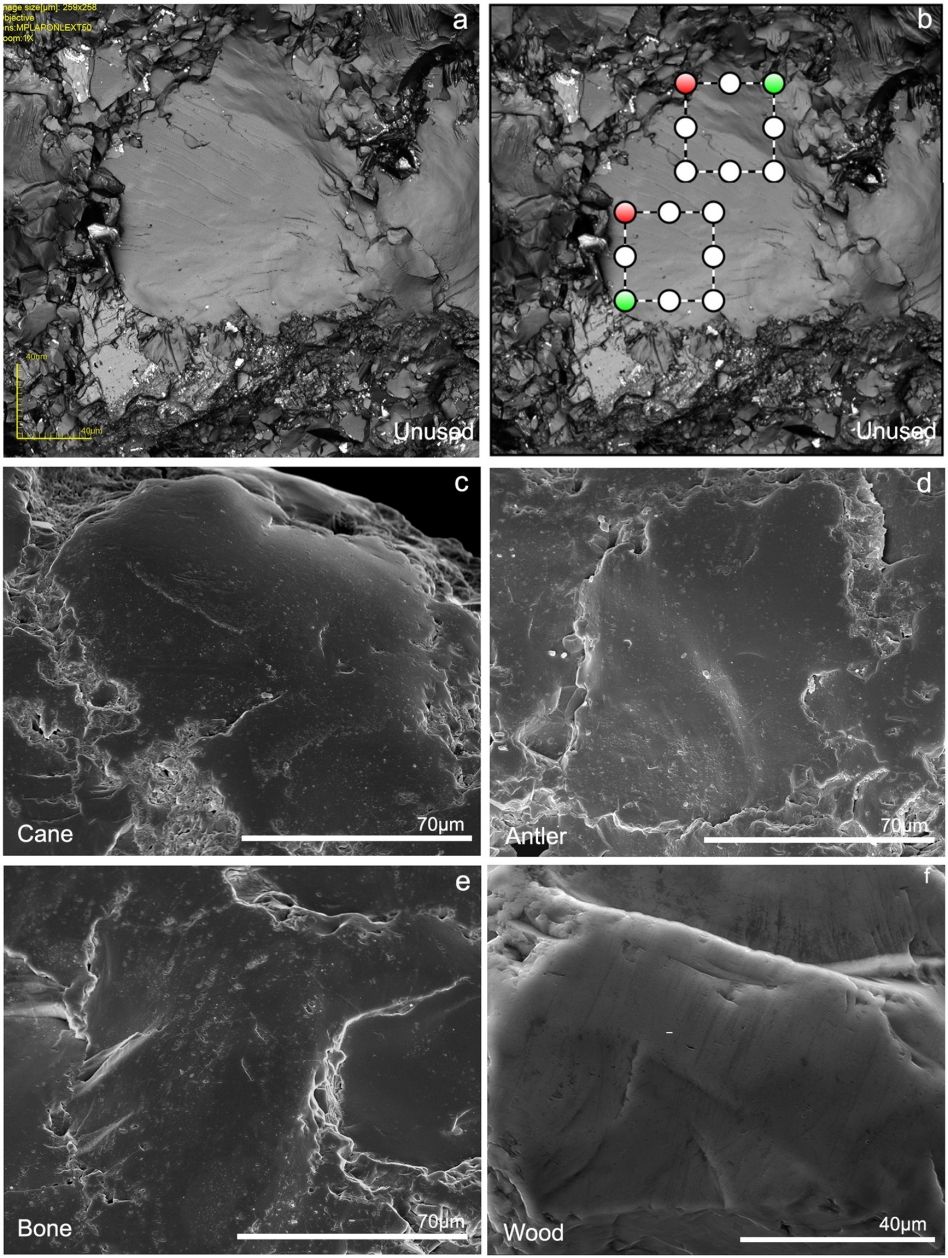
Well-developed polishes originated from contact with different materials and unused surfaces. a) LSM-maximum intensity image of an unused surface. Note the smoothness of the bigger quartz crystals; b) The two selected areas where LSCM measurements were performed on the same unused area; c) SEM image of a polished area after contact with cane; d) SEM image of a polished area after contact with antler; e) SEM image of a polished area after contact with bone; f) SEM image of a polished area after contact with wood.

Attempts to identify the worked material based on polished surfaces have been already performed on chert [7, 10, 20, 32, 39]. The resulting 3D surfaces were statistically sorted into different categories applying different statistical tests and the results generally pointed to a straightforward recognition of the worked materials. For example, the use of the roughness parameter *Rq* (root mean square roughness according to ASME B46.1 [2002], similar to the eponymous parameter in the ISO 4287 norm [64]) showed a remarkable capacity to discriminate polished areas on English flint used to work different worked materials (antler, hide, wood) [10: Fig 5]. Ibáñez et al. [32, 40] enlarged the range of worked materials, adding cereals and cane. Based on ISO 25178 parameters, discrimination of wild from domestic cereals worked better when large surfaces (200x200 μm) are considered [40], while analysis of smaller surfaces (50x50 μm) was insufficient to discriminate them [32]. In the latter study, the authors applied a statistical approach based on a decision tree model, similar to the present analysis. With this approach, they were able to correctly identify the worked material in 67% of the cases [32].

The only available studies on quantifying quartzite worn surfaces comprise relatively limited sample sizes (four samples, 12 measurements per sample; 2 sub-types of the same variety, i.e. Mistassini Quartzite) and only one contact material (hide) [22, 23]. Scale-sensitive fractal analysis (SSFA) was applied to discriminate between used and unused areas as well as between worn areas on tools used to work either fresh or dry hide. One algorithm that measured the relative area (*RelA* or *Srel*) was employed in the first study [22], while a different parameter, *Asfc* (area-scale fractal complexity, which is based on *Srel*) was tested in the most recent one [23]. Both parameters proved valid to discern used surfaces as well as to discriminate the two states of the one worked material included (fresh vs. dry). Moreover, the different grain-sizes of the two sub-types of quartzite affected the discrimination degree of the analyses at different scales, demonstrating that the degree of coarseness in coarse-grained materials has to be taken into account when quantifying irregular lithic surfaces. This probably applies to other lithic raw materials too.

In this study, we evaluated the potential of confocal microscopy to discern contact materials based on 3D measurements of polished areas formed on quartzite surfaces. Two different quartzite varieties were included for a total of twelve experimental flakes. Different surface parameters (n = 37: ISO 25178–2, SSFA, furrow analysis, and texture isotropy and direction analyses) were calculated to characterize worn surfaces of tools used to work different animal and vegetal materials (antler, bone, cane, skin, wood). Eight analyzed surfaces were identified as potentially problematic (Table 3). The issues stem mainly from the very rough topography of quartzite and its coarse-grained structure (see below), making this material particularly difficult to scan and analyze without artifacts. The statistical analysis was run on the full dataset and on the dataset excluding these surfaces. While the general results are comparable, the details vary greatly between the two analyses. All results are available on Zenodo (https://doi.org/10.5281/zenodo.4249219) but we have argued that the eight potentially problematic surfaces are representative of the difficulties of scanning and analyzing quartzite. Therefore, only the results for the full dataset are discussed below.

For the analysis, a sub-set of parameters with a high predictive power (*Vmc*, *Mean depth of furrows*, and *Mean density of furrows*) was selected and used to compare the same experimental polished surfaces. The experimental sample was divided into a training and a test set in order to check the validity of the selected parameters to identify the worked material through two classification models. Two different classifiers, a decision tree and a support-vector machine (SVM), were selected in order to assign every surface texture to a worked material based on a selected number of parameters (i.e. features). The decision tree was of great interest because of its ease in interpretation and accessibility without the need to run codes. The SVM was chosen as a more capable algorithm to check if the results are limited by the capacity of the decision-tree models, which is not the case here, as shown by the similar performance with the decision tree model. Due to the limited number of samples, the predictive performance is expected to profit most strongly from further data points. Other machine learning algorithms that rely even further on large training data, such as deep neural networks, are considered inappropriate for the analysis at hand, despite a high potential performance.

Results of the measurements performed on the surfaces of the experimental quartzite flakes presented in this preliminary study are very promising and can be used to develop further research. Considering the limited dataset (twelve flakes, 86 sub-areas), results provided by the decision tree classifier excluding quartzite type as a feature allowed a 47% rate of discrimination of polishes originated from contact with five different materials. The discrimination of bone and hide polishes shows 100% of correct identifications, although the identification of the state of the hide (fresh vs. dry) was not successful, as observed in other studies on chert [32]. This result contrasts with what was observed on quartzite samples by Stemp et al. [22, 23], as they were able to discriminate the state of the worked material. Wood and antler show partially overlapping values; therefore, they are sometimes grouped together (40% of antler misidentified as wood and 33% of wood misidentified as antler). In 20% of the cases, antler is incorrectly classified as bone. Considering that antler and bone are also difficult to distinguish in conventional use-wear studies [17, 18, 88, 89], it can be that the marked similarities of worn surfaces resulting from contact with these materials are due to their very similar material properties [90].

Polish on tools used to work cane appears problematic, as only 40% of the cases are correctly classified. Cane polish has also been incorrectly assigned to unused and antler (40% and 20% respectively). Unused surfaces are also complicated because they are equally assigned to antler, unused or wood (each 33%). This seems to be an issue related to the quartzite varieties included in this study, as unused English flints could be discriminated from used samples [10].

### Polish formation on quartzite and quantification

Quartzite and chert are both composed mainly of silica (> 90–95%). The main microscopic difference observed between the two big rock categories is noticeably the relative grain size [16, 91]. Quartz crystals in quartzite typically range from 30 to 150 μm, while in chert they measure only 3 to 10 μm. This significantly affects the detection of wear features, especially of polish. Generally unused chert surfaces under a microscope are seen as single silica grains packed together, forming a relatively homogeneous surface. The abrasion-driven polishing process on chert, as well as on other fine-grained raw materials, is visually perceived as a transition from a relatively rougher surface to a significantly smoother area. Due to the fact that the original surfaces are much more regular, therefore flatter, on chert than on coarse-grained materials, large polished areas are visually perceived at fine scales as very flat and smooth under reflected light microscopy, which makes them generally very bright [17, 18]. The difference in micro-topography between raw materials explains also why polish forms relatively faster on fine-grained materials than on coarse-grained ones. The highest parts of the topography (i.e. peaks) are always abraded first, so polish starts to develop there. Yet, differences in height between valleys and hills on chert are so negligible, probably due to the small granulometry, that polish can propagate quickly across the surface. As well as being larger, typical quartz grains in quartzite (and other coarse grained raw material) can have different orientations, creating further variation in surface relief compared to chert. This variation in orientation also affects their visualization with optical microscopy, with SEM allowing for easier distinguishment of the grain borders. Such borders can be obliterated in advanced stages of polish formation [41, 43].

This is the main reason why, no matter the worked material, polish is generally limited to the highest topographical parts (i.e. hills) on quartzite and rarely affects the valleys in between [58]: since the height differences between valleys and hills are greater and hills are much sparser on quartzite than on chert (due to grain size and orientation), polish cannot propagate on quartzite as much as it does on fine-grained materials (during comparatively similar time spans).

This can have major implications on the acquisition process with confocal microscopy, affecting mainly the general size of the acquired surfaces [22, 23, 36]. In other words, the fact that well-developed polish on chert can cover areas as big as *ca*. 200x200 μm [32], and sometimes a lot larger, has important effects on the selection of sub-areas to be analyzed with confocal microscopy, and therefore on wear quantification itself.

When confocal acquisitions are performed on quartzite, relatively small surface areas need to be considered (50x50 μm) if one aims at sampling only polished surfaces, thereby ignoring the unpolished ones. Such surfaces generally coincide with the interior of quartz grains, where polish is easily detectable (Fig 5b and S2 Fig). As mentioned earlier, even such small areas can cover more than single grains and can be difficult to scan and analyze.

**Fig 6:**
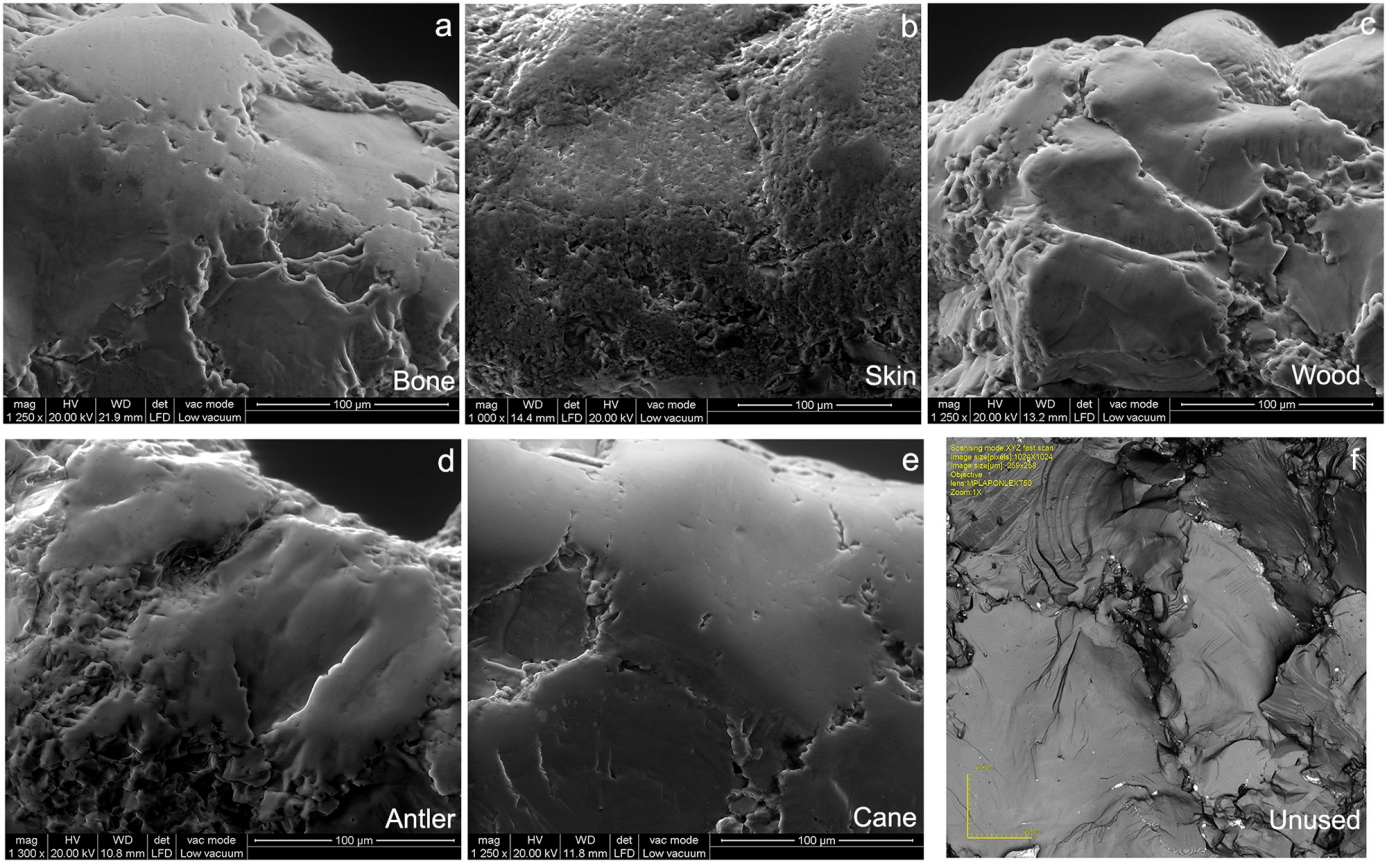
Close-ups of well-developed polished areas (FOV≈200μm). a) SEM image of a polished area after contact with bone; b) SEM image of a polished area after contact with dry skin; c) SEM image of a polished area after contact with wood; d) SEM image of a polished area after contact with antler; e) SEM image of a polished area after contact with cane; f) LSM-maximum intensity image of an unused surface.

**Fig 7:**
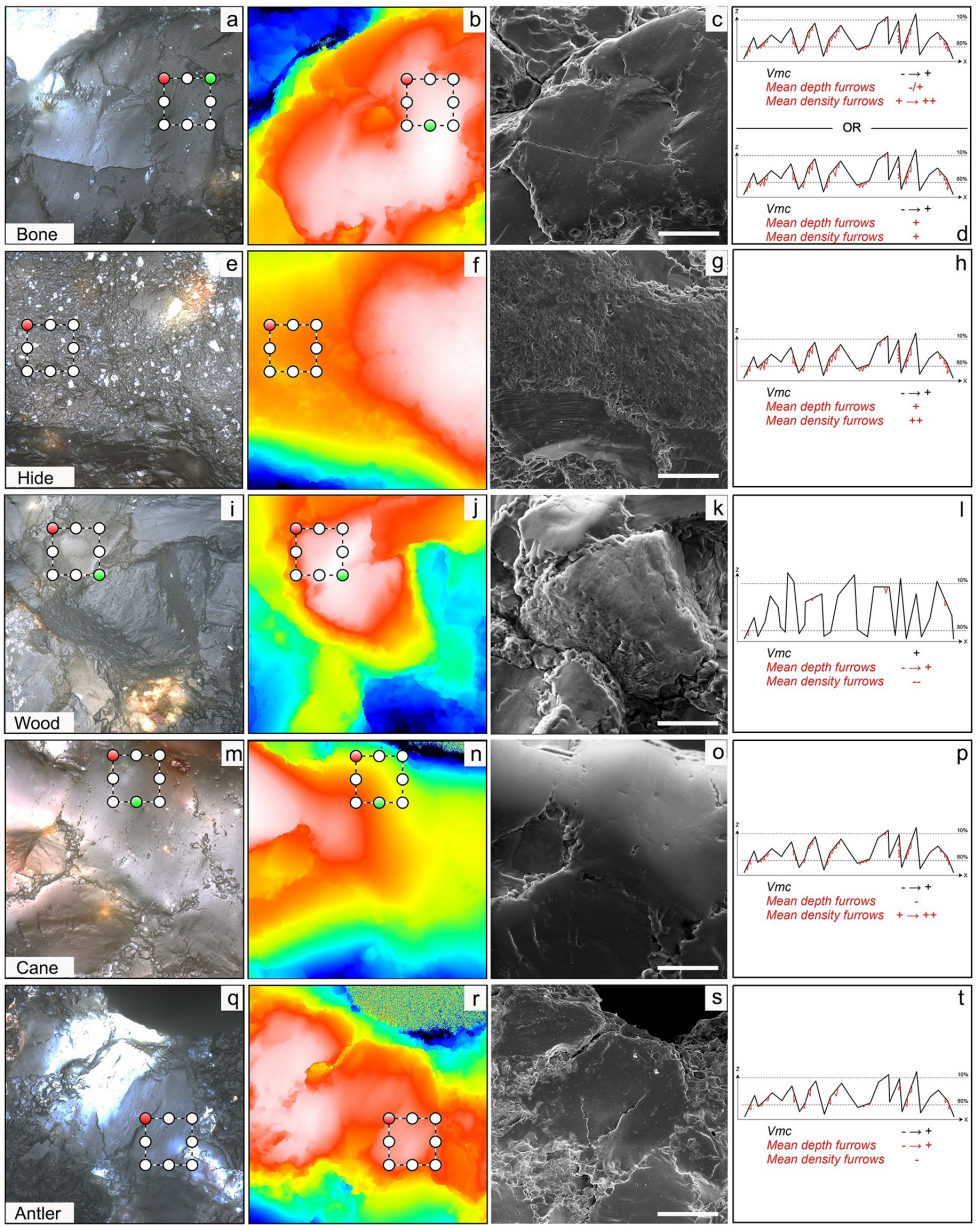
Bright field images (left), topographies (middle-left), SEM images (middle-right) and schematic representations (right) of the textures characteristic (based on the decision tree; Fig 4) of the different worked materials. For simplicity, the schemes show X-Z profiles, in black, with furrows superimposed in red. *Vmc* is calculated as the volume below the surface (= area below the XZ profile) and between the dashed lines. Note that the scales are not preserved and that the values are to be considered only in relative terms among the schemes. When a range is given for a parameter (e.g. "- → +" for *Vmc* on antler), it means that the values can vary within the given range. When the range include the extreme values (e.g. "- → +" for *Vmc*), it means that this parameter is not characteristic for this worked material. See Table 4 for the values of the selected parameters for each surface.

When unused samples are analyzed, same sized scans are acquired on single quartz grains, which appear very smooth (Fig 5a and 5b and S11a Fig) and texturally similar to polished grains (Fig 5c–5f). The low *Vmc* values and the low density of furrows confirms this (S11c Fig). Only when polish is particularly well-developed, the borders between grains start disappearing, forming more homogeneous surfaces (Fig 6a and 6e). The fact that the visual texture of unused and polished crystals (only when smooth polish forms) is visually alike (Figs 5 and 6) could help explain why in this study it was hard to discriminate unused samples (Fig 5a) from antler and wood polishes (Figs 5d, 5f, 6c, 6d, 7i–7l and 7q–7t).

When the three parameters selected in this study are plotted one against the other in pairs (S12 Fig), considerable overlaps among various worked materials are evident. Nevertheless, some worked materials occupy non-overlapping spaces in the plots, and this mirrors the results of the decision tree. The decision tree classified surfaces with high *Vmc* and very low density of furrows as wood-working (Fig 7l; Table 4), while surfaces with low density of furrows were classified as having worked antler (Fig 7t); remember, however, that some of these surfaces were wrongly classified, explaining the large overlap on the bivariate plot (S12b Fig). For the same reason, the extremely smooth cane polish (Figs 5c, 6e and 7m–7o; Table 4), characterized by lots of shallow furrows (Fig 7p), has been wrongly classified as either unused or antler in some instances. Conversely, bone proved to bear clear indicators picked up by the textural parameters used in this study that allowed a perfect classification, despite the small dataset, based on the numerous deep furrows (Fig 7d), although not as extreme as hide textures (Fig 7h and S12a Fig). This could be related to the fact that bone polish (Fig 7a and 7c), in addition to being extremely smooth, regularly displays other types of use-wear, such as striations and pits (Figs 5e and 6a) [17]. These, and other so far unknown features, could explain the high degree of classifications for this worked material type.

**Table 4:** Values of the selected features for the surfaces shown in Fig 7 and S11 Fig.

Sample	Point	Area	Material	*Mean depth of furrows* (nm)	*Vmc* (μm^3^/μm^2^)	*Mean density of furrows* (cm/cm^2^)
VSH4-5	3	2	Bone	139.17	0.11	2350.70
VSH4-3	2b	1	Hide	365.22	0.26	2524.55
VSH4-4	1a	1	Wood	487.85	0.73	2033.89
VSH4-2	2b	2	Cane	61.19	0.12	2439.83
VSH4-1	1a	2	Antler	252,17	0,41	2149.65
A35-5	2	1	Unused	102,66	0,08	1611.55

A possible solution to overcome these shortcomings could be analyzing larger areas including more quartz grains (e.g. *ca*. 200x200 μm) (Fig 6), as done for chert in Ibáñez et al. [32]. This would first allow better characterization of the unused surfaces on quartzite (Fig 6f) by quantifying textural topography including the borders between grains. In fact, considering larger areas could potentially overcome the issue that emerged in this study when unused surfaces were mistaken by used ones (antler, wood). Along with helping in solving this problem, the simplistic dichotomy of smooth vs. rough polished surfaces generally employed in conventional studies to describe visual polish appearance (Fig 6a and 6b) could be replaced by quantitative descriptors. However, it should be reminded that particularly steep surfaces are difficult to scan and analyze. When such surfaces are being scanned specific adjustments to the acquisition and analysis settings might be required.

It is possible that applying the same parameters on larger acquired 3D surfaces could take additional features into consideration, such as variability of polished hills and valleys (Fig 6c), state of the borders in between the grains (Fig 6a–6e), undulation traits (Fig 6d), and pits (Fig 6a and 6e). All these visual features are somehow missed when smaller surfaces are acquired and analyzed. Additionally, polish is characteristic of more advanced abrasion processes, but non-polished areas do not have to be necessarily in an unused state. These areas might have been in contact with the worked material for shorter amounts of time but might still have a texture different from that of truly unused areas. By considering all these aspects, more accurate probability statements for quartzite could be provided.

Another way to better understand the polish formation on quartzite and the gradual textural changes (from smooth original quartz grains to “smoother” polished surfaces) could be to set up controlled sequential experiments [92, 93] integrated in quantitative wear studies. One interesting development could be integrating the sequential observations with quantification of the same surface area throughout time by using the original coordinate system presented in Calandra et al. [94].

The fact that the raw material type seems not to have a major influence on our results [as observed for chert by 32] is a very useful insight for planning future experiments. Nonetheless, we think that more varieties of quartzite should be tested in the future to improve the classification power of the model tested in this study. This data is apparently in contrast with what was observed by Stemp et al. [22, 23] on the Mistassini variety, where significant variability was documented between the two types. Moreover, discrimination of used vs. unused surfaces on this quartzite variety as well as between samples used to scrape dry vs. fresh hide, were understood to be highly dependent on the scale of analysis, mainly due to different granulometry. The noticeable difference between Stemp et al. study and ours is the objective used to acquire 3D data and the parameters used to discriminate the surfaces. Stemp et al. used a 20x (NA = 0.60) objective, while we used a 50x (NA = 0.95) one. They opted for testing single parameters (*Srel* or *Asfc*), while we tested 37 parameters and then selected a sub-set of them (three). Major differences in the results, mainly the capacity of discerning unused surfaces, could be explained by the acquisition scale. It might well be that used vs. unused surfaces on coarse-grained materials are better discriminated when analyzing larger surfaces acquired at lower magnifications [22, 23]. On the other hand, the very high classification rates of our analysis on specific polishes (contact with bone and skin), demonstrates that surfaces acquired with a 50x objective do have the potential of accurately classifying some materials.

Considering all this, more experiments including larger samples (more quartzite types, more worked materials, more samples per worked material, more scans and larger areas per sample) are needed to better characterize worn areas on quartzite and to increase the accuracy of the model presented here. Moreover, comparison between studies should be performed in the future in the attempt to understand the best combinations of acquisition and analysis settings and of different parameters for a proper quantification of use-wear on quartzite.

## Conclusion

Although conventional inspections of use-wear on archaeological assemblages have been frequently employed and led to interesting insights about how stone tools were used in the past, criticisms have been raised and they still persist. They mostly revolved around the subjectivity of the analyst always present in all functional interpretations and the semantical divergences of the descriptions of wear provided by different specialists. Due to the general lack of quantitative descriptors in conventional functional interpretations, it is extremely hard to objectively compare use-wear databases built by different researchers.

On the other hand, limitations of the quantitative approach, as it is presently being developed, also concern data comparison. In fact, different techniques and different pieces of equipment to acquire 3D surface data are employed. Moreover, few and different parameters are selected and different analytical workflows (with different cut-offs) are also systematically used. All this makes it difficult to meaningfully compare published quantitative data.

This study, as several others, is an effort to fill in this gap by systematically applying well-recognized methods to objectively describe surface modifications on stone tools. Even though our results are preliminary, mainly because of the small sample used, the quantitative data we report in this paper add to previous studies indicating that confocal microscopy will likely be a key approach for the future development of the method. By using LSCM, large datasets of worn surfaces on different raw materials could be compiled and shared between researchers. The numerical descriptions of the processed 3D surfaces have the potential to overcome two of the most critical issues of traceology behind researchers’ skepticism including subjectivity of interpretation and different terminology employed to describe use-wear [37].

Furthermore, once large experimental datasets are available for comparison, confocal microscopy will become an increasingly viable mean of assessing archaeological assemblages for determining the contact material, if polished areas are available. However, possible obstacles of the systematic application of this technique to distinguish contact materials based on worn surfaces is the effect that post-depositional surface modifications (PDSMs) might have on its accuracy [95, 96]. Hence, more experiments involving trampling and sediment contact are necessary for further development of the method.

Although the data presented here are preliminary, they allow for a better understanding of polish appearance and formation on quartzite. This study not only proves that quantitative methods are a valid tool to differentiate polished areas originated through contact with several materials (animal and vegetal ones) on quartzite tools, but it also provides thorough datasets (with raw data) of the surface texture measurements acquired on the experimental replicas (see Data Availability below). Therefore, all data are fully accessible, and different surface analysis workflows and statistical comparisons will be possible with future research. Moreover, the full reproducibility of data acquisition is guaranteed by the reporting of the acquisition settings of the microscope used in this paper [37, 38].

In sum, this study highlights the great potential of confocal microscopy to solve the ever-lasting debate on the accuracy of functional interpretation of use-wear based on visual attributes and it stands as a bridge towards the systematic application of metrology in the analysis of use-wear. We hope that our data will constitute a step forward towards the integration of quantification methods in conventional microscopic examination of use-wear on stone tools. Moreover, we hope it can be used to increase the reproducibility and comparability of use-wear data acquired by different researchers.

## Supporting information

S1 Fig The outputs of any confocal measurement and analysis workflow. a) WF image; b) Maximum intensity map; c) Height map (topography); d&e) Maximum intensity and height maps of the first sub-area before processing; f&g) Maximum intensity and height maps of the second sub-area before processing; h) Analysis workflow using Confomap; i) Height map of the first sub-area after processing; j) Height map of the second sub-area after processing.
(TIF)

S2 Fig Examples of the locations of the two extracted surfaces (sub-areas) on polished surfaces originated after contact with different worked materials. Variation on the two different varieties can be compared. a&b) Antler; c) Bone; d&e) Cane; f&g) Wood; h&i) Cane; l&m) Wood; n) Dry skin; o&p) Fresh skin.
(TIF)

S3 Fig Mutual information on training set with type. (PDF)

S4 Fig Working principle of the classification algorithms. An example dataset with three classes (“blue”,”gray”,”red”) and two features (x and y axes) is shown in both plots, where each dot represents a data point. The classification algorithms aim to segment the plane into areas, in which ideally only data points of one class exist. One main difference is the type of boundary line that is allowed for a segment. The decision tree classifier for instance is restricted to boundaries parallel to the feature axes, while the SVM may use curved boundary shapes.
(PDF)

S5 Fig Comparison of the performance in the classification rate when some variables are either considered or discarded. The different possibilities depend on whether all the features or the selected set of features are used and whether the type of quartzite is considered as a feature or not.
(PDF)

S6 Fig Normalized confusion matrix on the test set for SVM when the type of quartzite is considered as a feature. (PDF)

S7 Fig Normalized confusion matrix on the test set for SVM1 when the type of quartzite is not considered as a feature. (PDF)

S8 Fig Normalized confusion matrix on the test set for SVM2 when the type of quartzite is not considered as a feature. (PDF)

S9 Fig Normalized confusion matrix on the test set for SVM3 when the type of quartzite is not considered as a feature. (PDF)

S10 Fig Decision-tree model including the quartzite variety (type). (PDF)

S11 Fig Maximum intensity map (left), topography (middle) and schematic representation of unused textures (based on the decision tree; Fig 4). See Fig 7 for details and Table 4 for parameter values.
(TIF)

S12 Fig Pairwise bivariate plots of the three selected features (*Vmc*, *Mean depth of furrows* and *Mean density of furrows*). The large dots represent the mean of each group and the small dots mark every measurement. The polygons are convex hulls.
(PDF)
