# Supplementary figures and images for: Polish is quantitatively different on quartzite flakes used on different worked materials
Source: PLoS One. 2020 Dec 3;15(12):e0243295. doi: 10.1371/journal.pone.0243295 (PMC7714215; doi:10.1371/journal.pone.0243295)

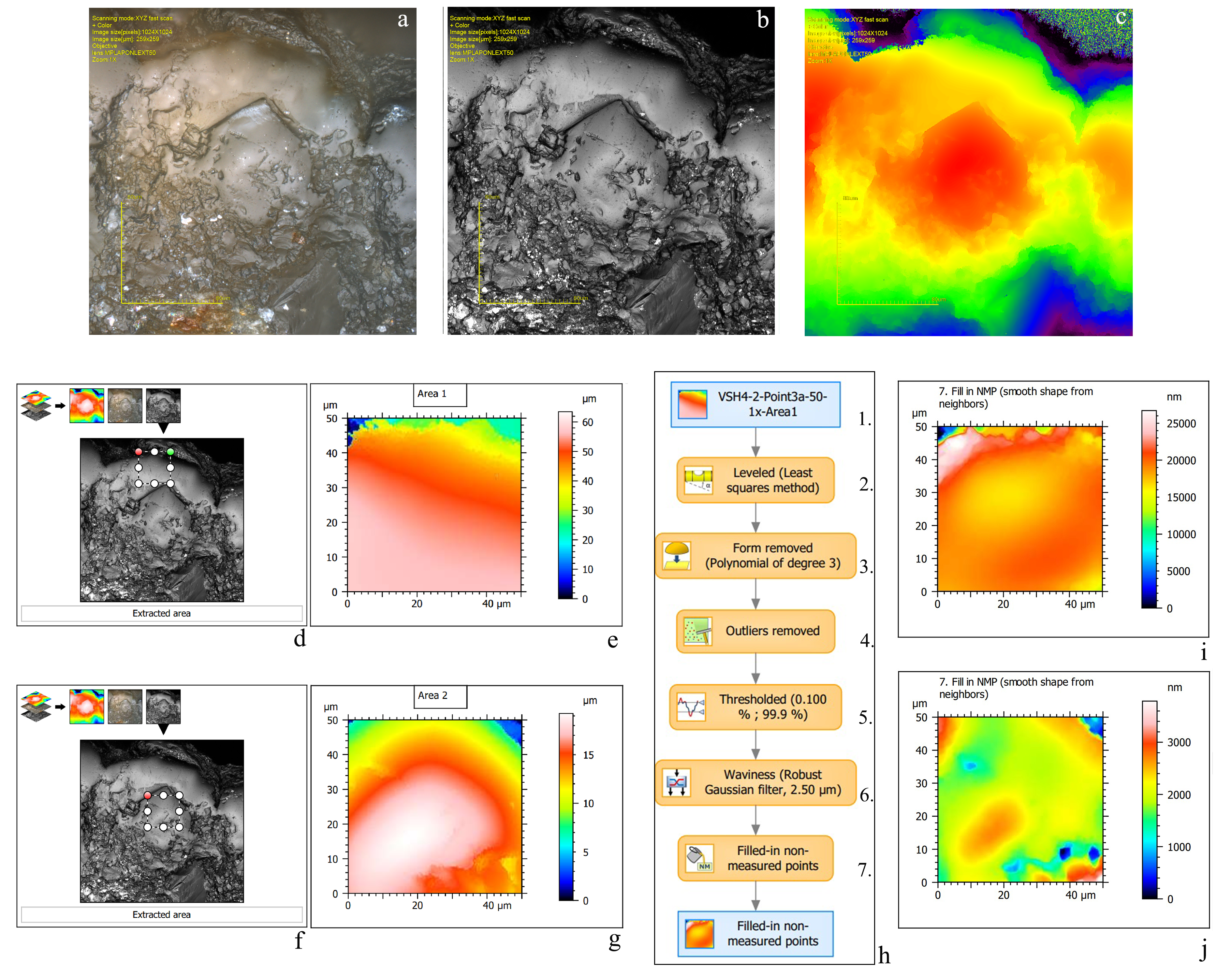

Supplement: S1 Fig — a) WF image; b) Maximum intensity map; c) Height map (topography); d&e) Maximum intensity and height maps of the first sub-area before processing; f&g) Maximum intensity and height maps of the second sub-area before processing; h) Analysis workflow using Confomap; i) Height map of the first sub-area after processing; j) Height map of the second sub-area after processing. (TIF) [file pone.0243295.s001.tif]

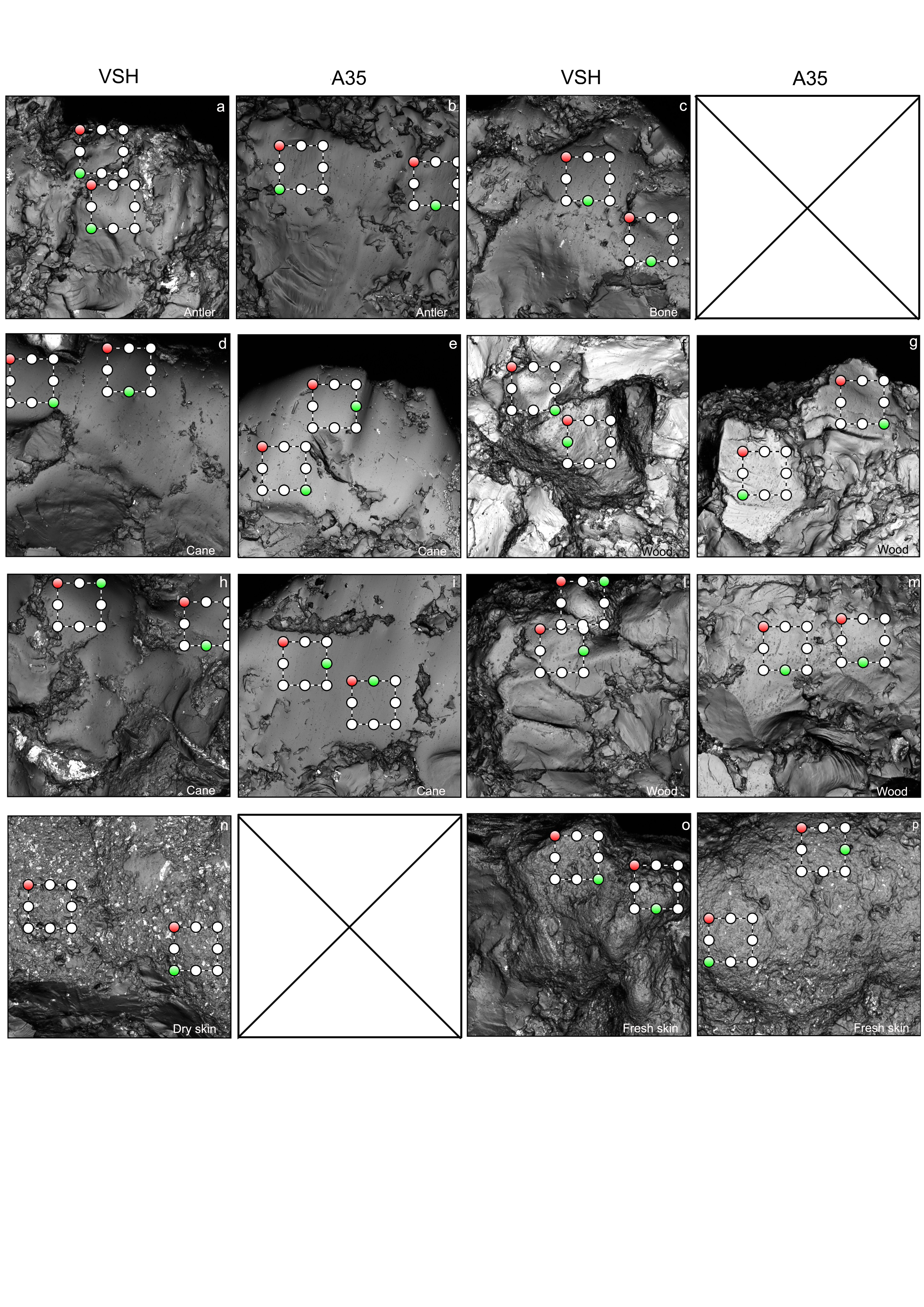

Supplement: S2 Fig — Variation on the two different varieties can be compared. a&b) Antler; c) Bone; d&e) Cane; f&g) Wood; h&i) Cane; l&m) Wood; n) Dry skin; o&p) Fresh skin. (TIF) [file pone.0243295.s002.tif]

# Mutual information on training set

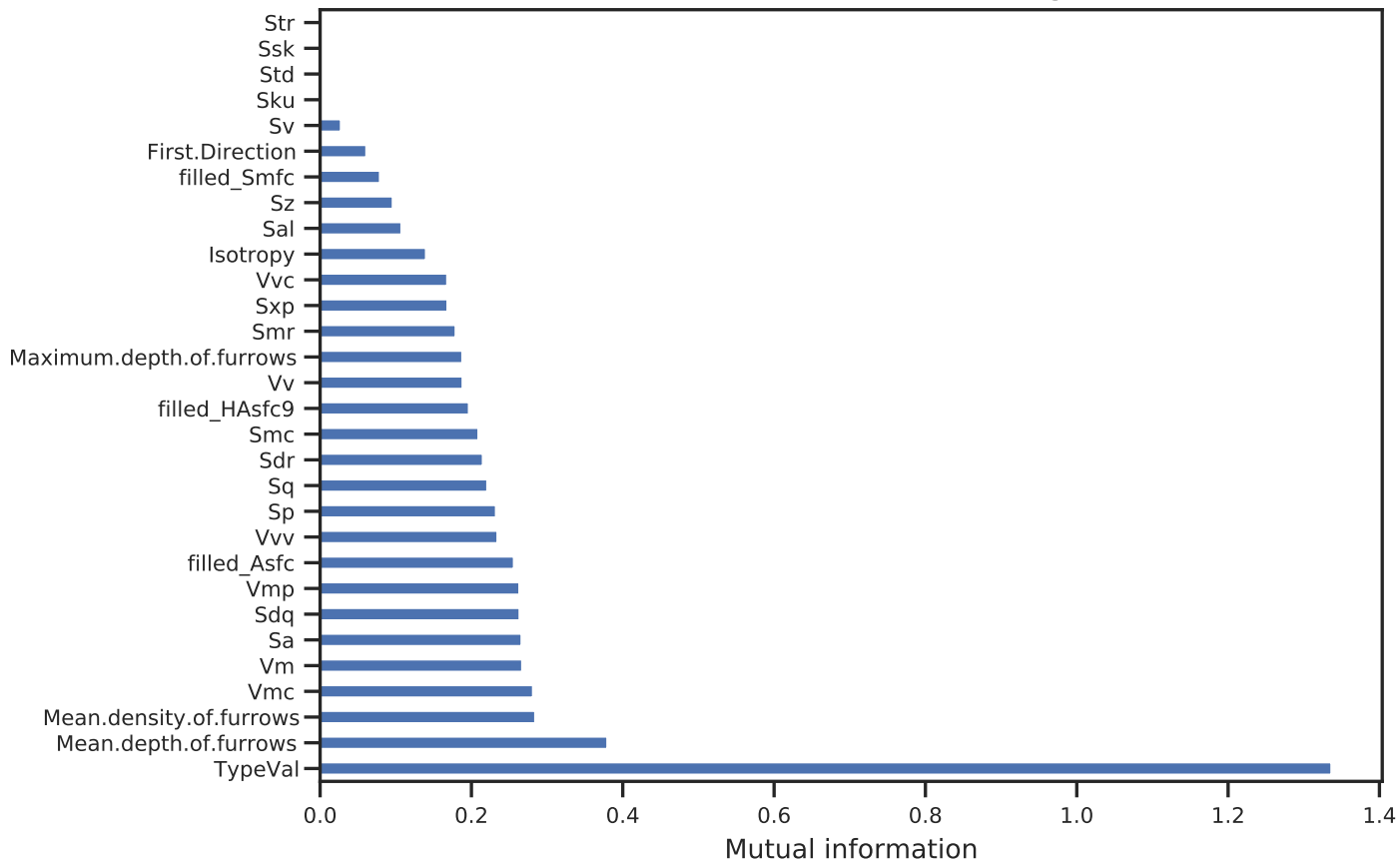

Supplement: S3 Fig — (PDF) [file pone.0243295.s003.pdf]

Decision tree

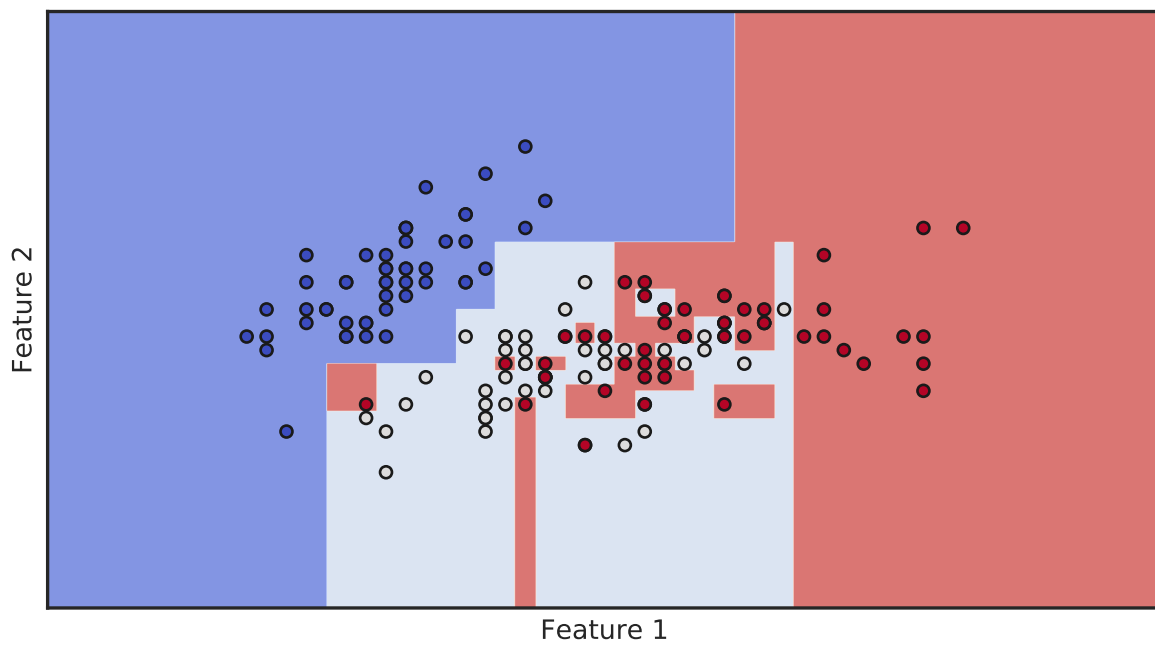

SVM with nonlinear kernel

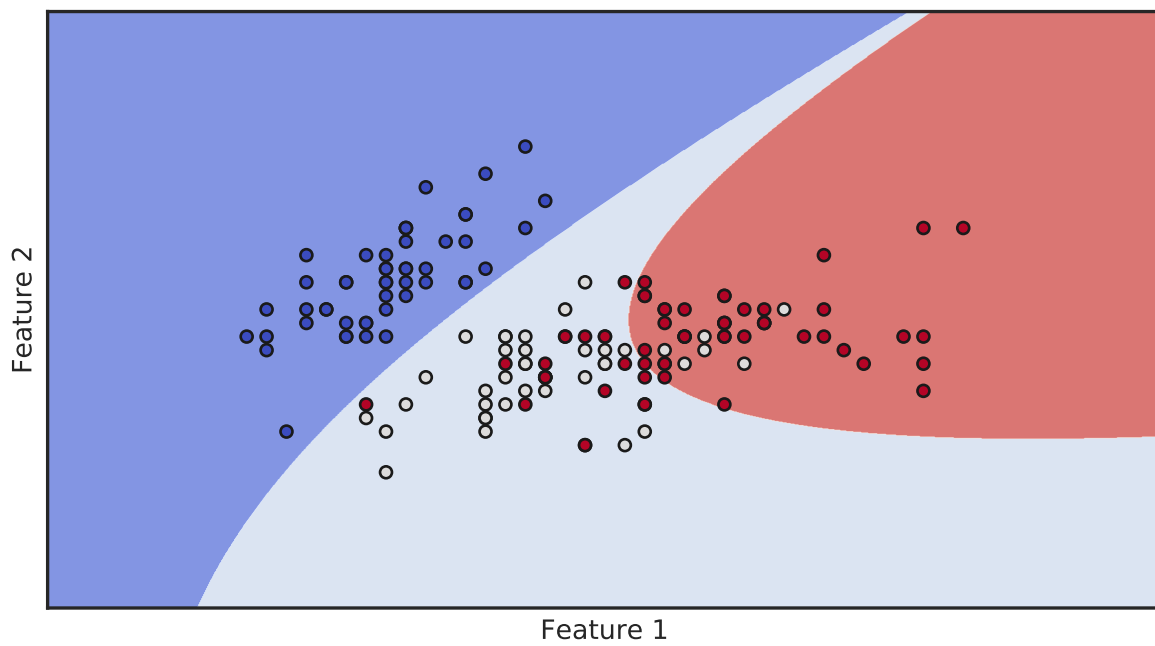

Supplement: S4 Fig — An example dataset with three classes (“blue”,”gray”,”red”) and two features (x and y axes) is shown in both plots, where each dot represents a data point. The classification algorithms aim to segment the plane into areas, in which ideally only data points of one class exist. One main difference is the type of boundary line that is allowed for a segment. The decision tree classifier for instance is restricted to boundaries parallel to the feature axes, while the SVM may use curved boundary shapes. (PDF) [file pone.0243295.s004.pdf]

Performance on training set

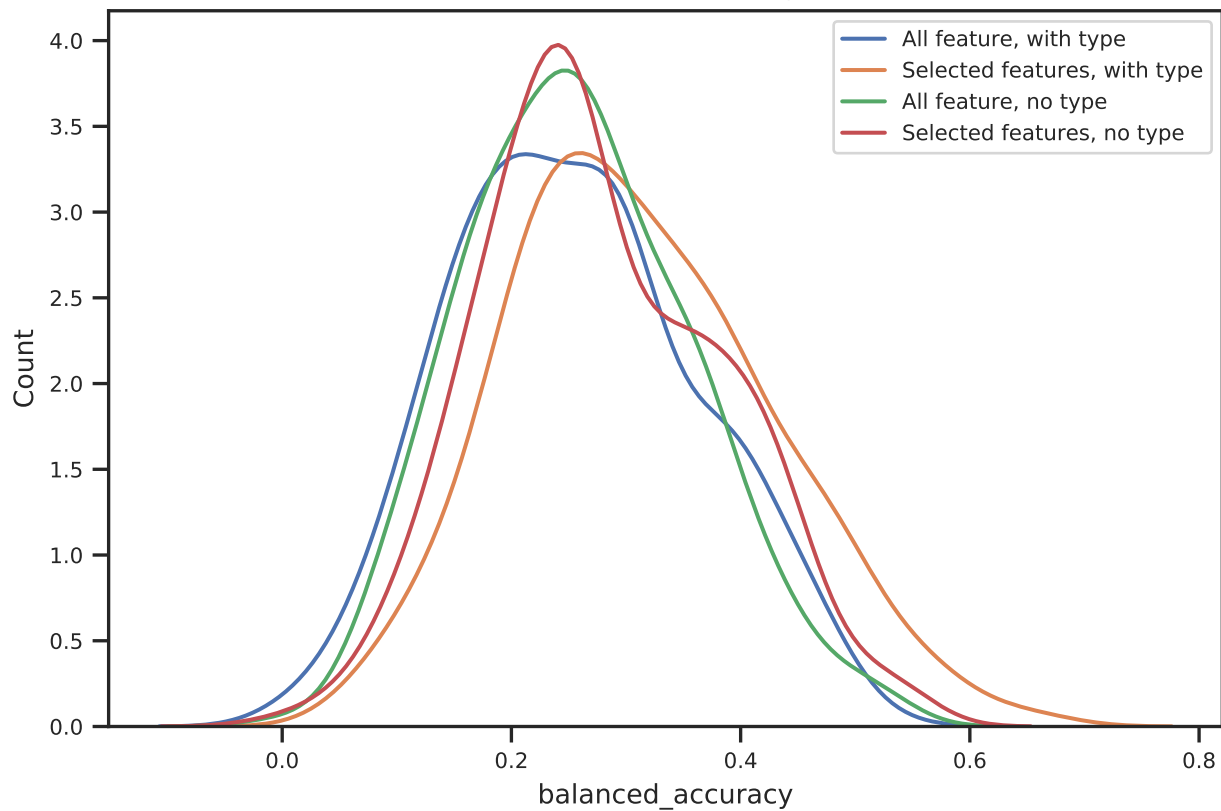

Supplement: S5 Fig — The different possibilities depend on whether all the features or the selected set of features are used and whether the type of quartzite is considered as a feature or not. (PDF) [file pone.0243295.s005.pdf]

Normalized confusion matrix on test for SVM with type

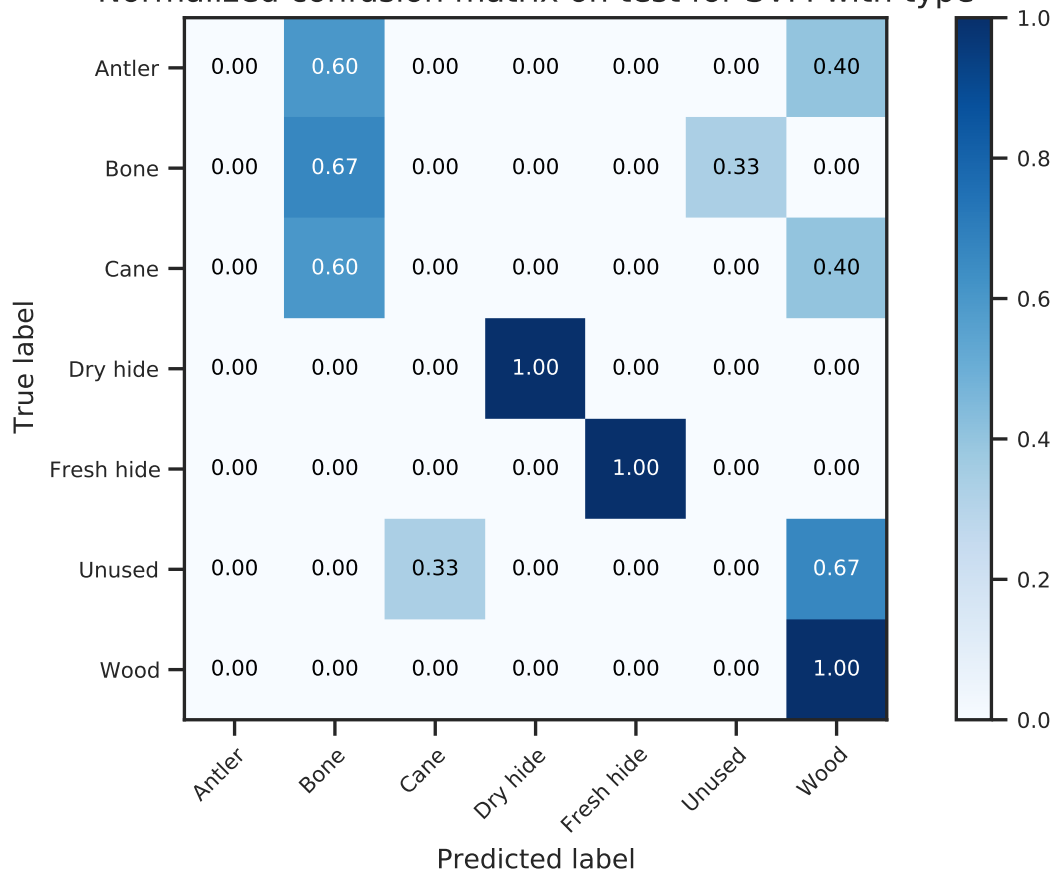

Supplement: S6 Fig — (PDF) [file pone.0243295.s006.pdf]

Normalized confusion matrix on test for SVM1 without type

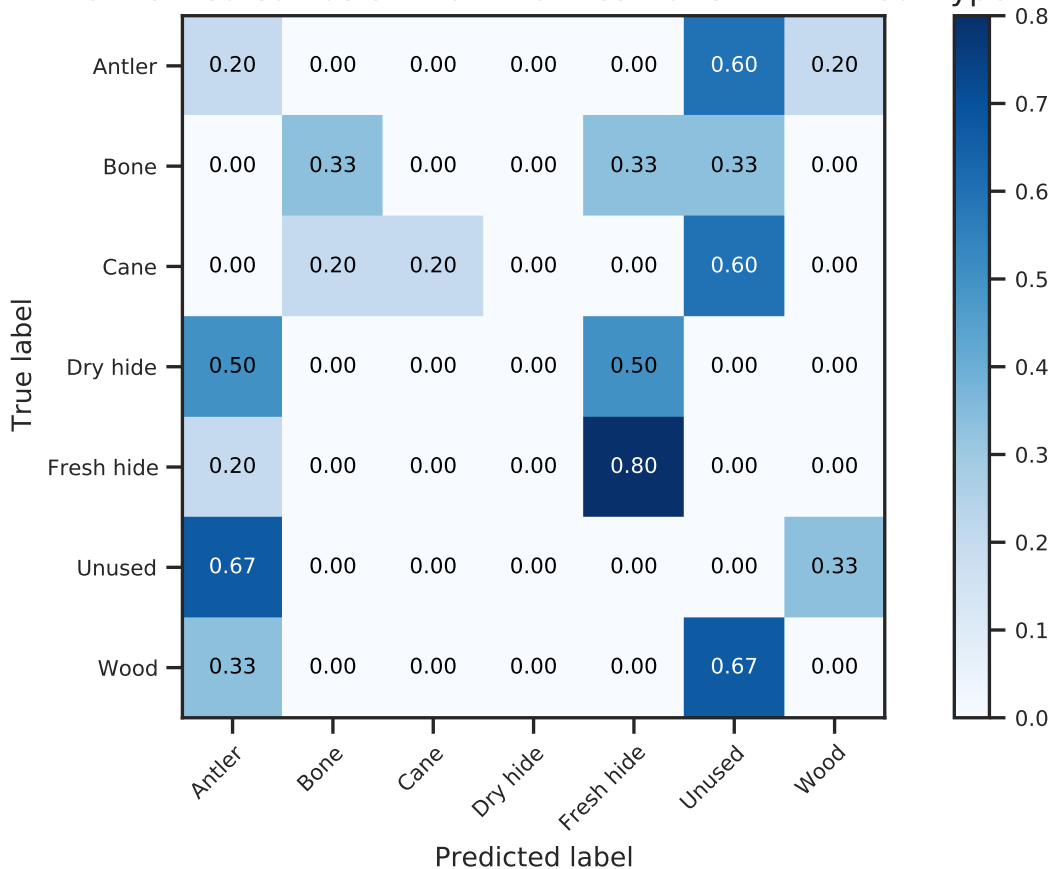

Supplement: S7 Fig — (PDF) [file pone.0243295.s007.pdf]

Normalized confusion matrix on test for SVM2 without type

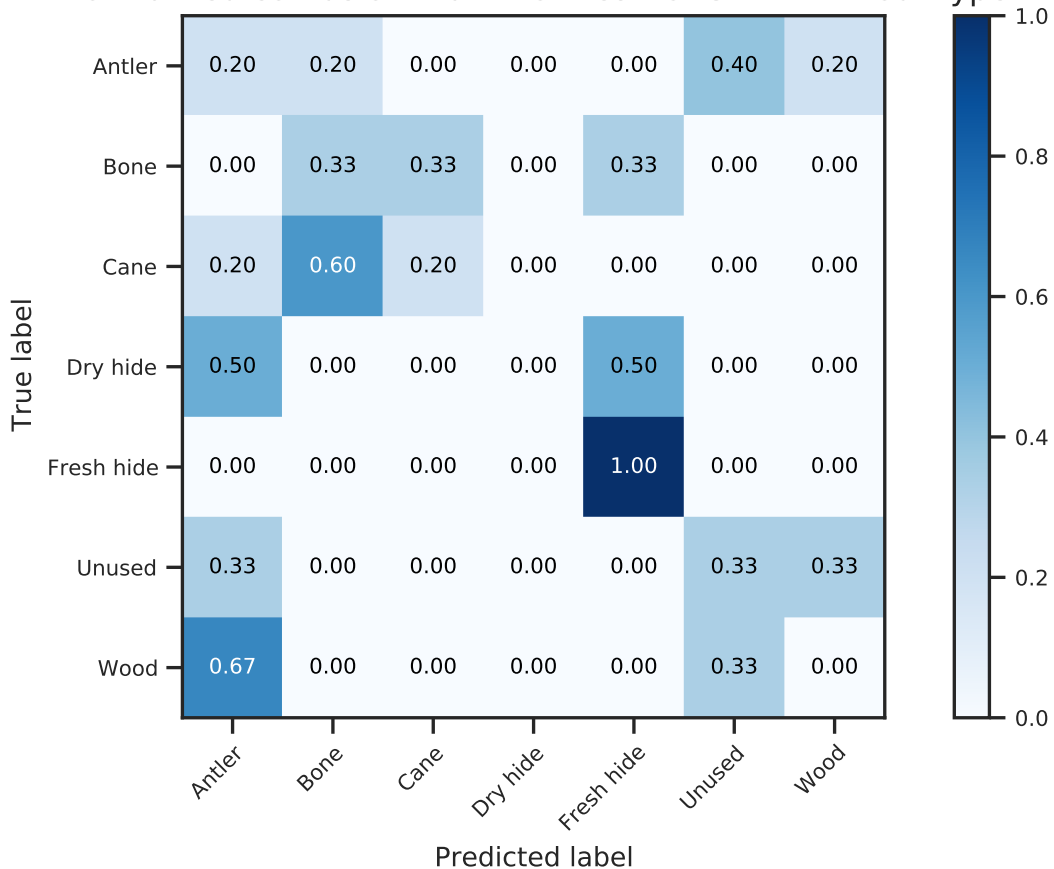

Supplement: S8 Fig — (PDF) [file pone.0243295.s008.pdf]

Normalized confusion matrix on test for SVM3 without type

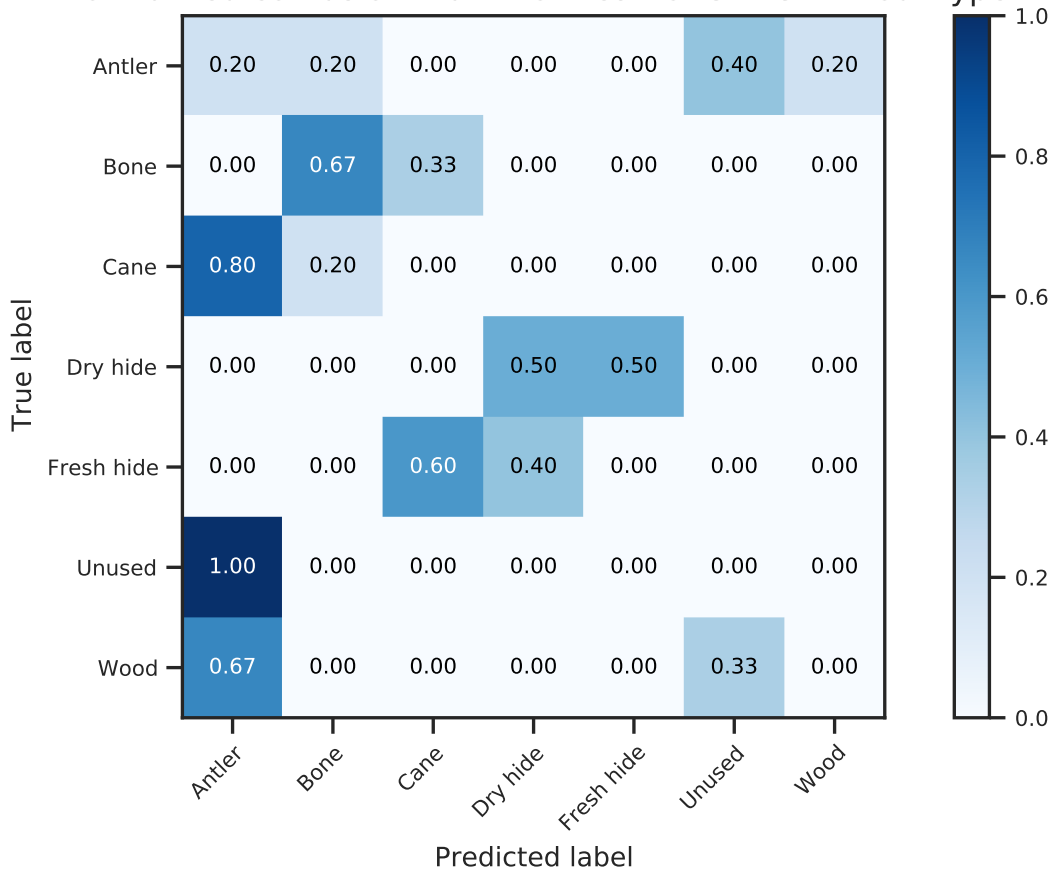

Supplement: S9 Fig — (PDF) [file pone.0243295.s009.pdf]

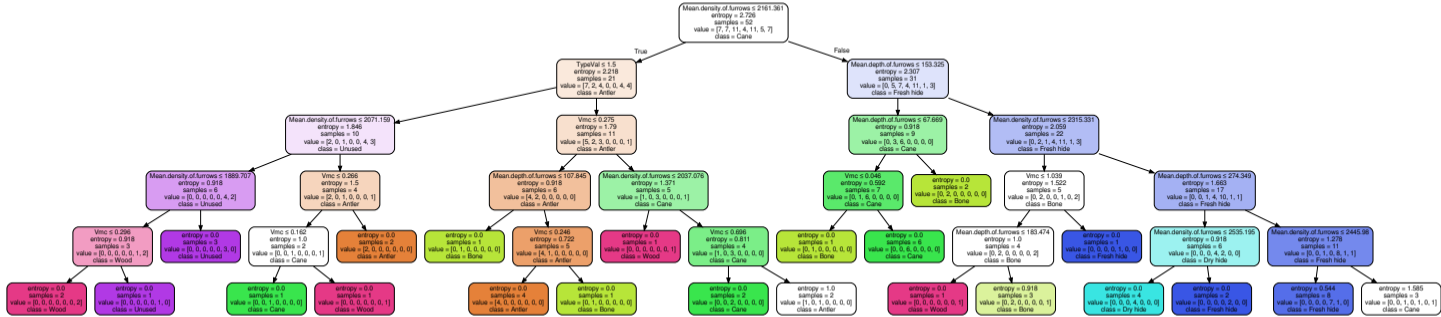

Supplement: S10 Fig — (PDF) [file pone.0243295.s010.pdf]

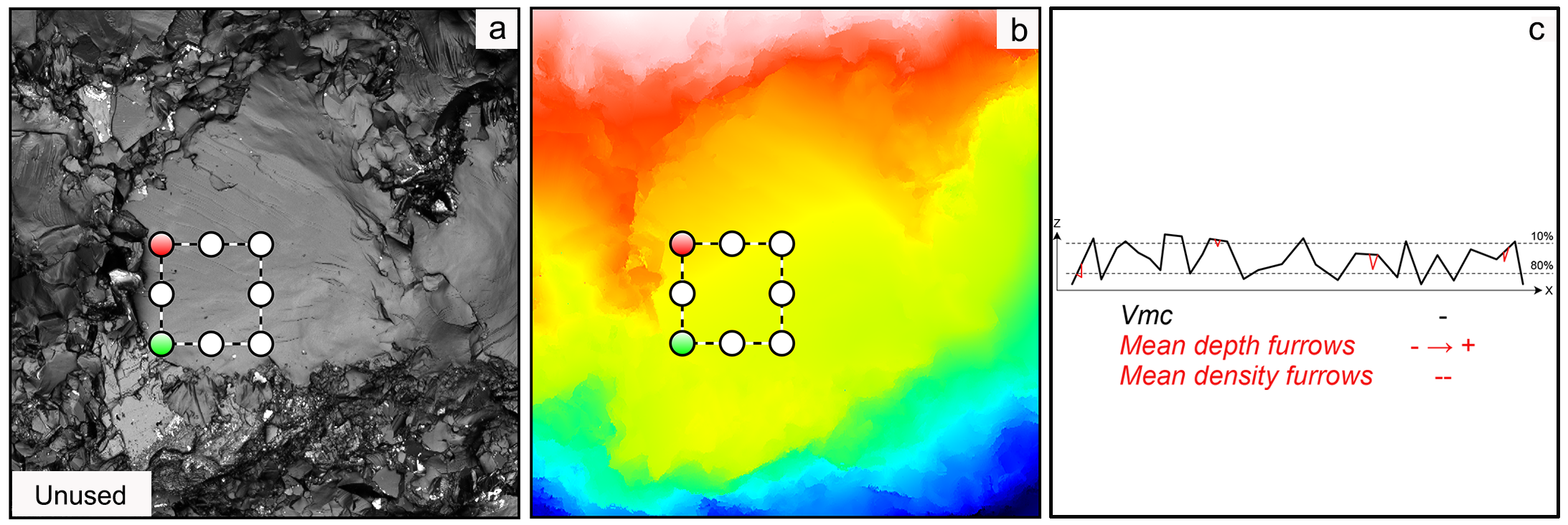

Supplement: S11 Fig — See Fig 7 for details and Table 4 for parameter values. (TIF) [file pone.0243295.s011.tif]

**a**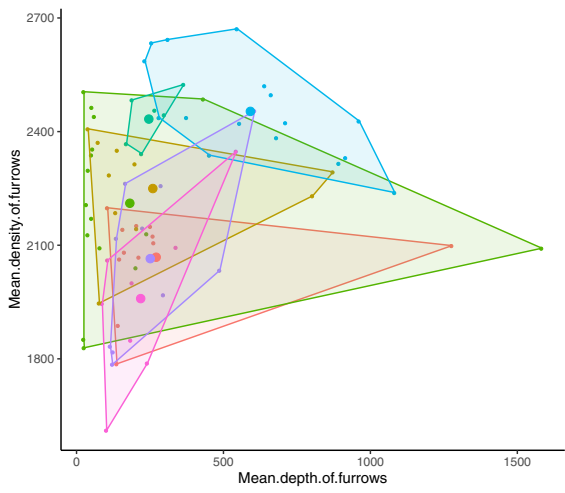**b**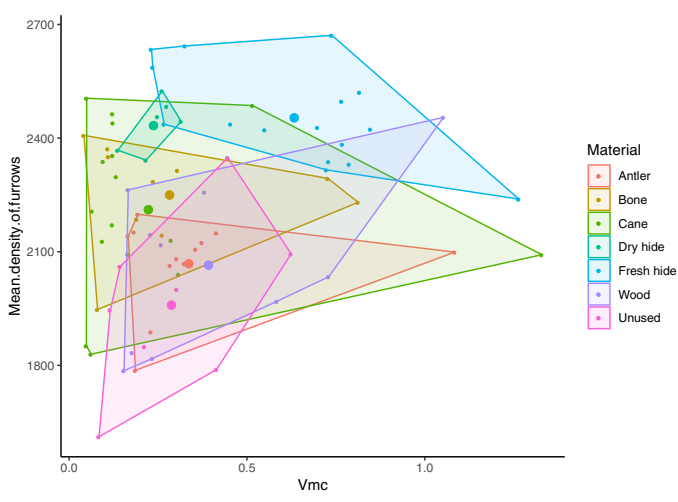**c**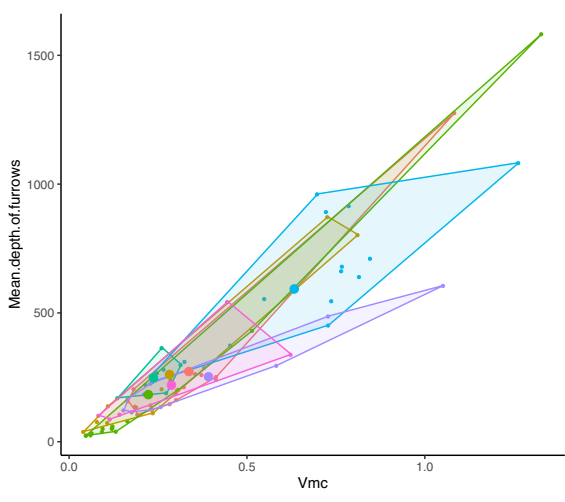

Supplement: S12 Fig — The large dots represent the mean of each group and the small dots mark every measurement. The polygons are convex hulls. (PDF) [file pone.0243295.s012.pdf]
